# Fast uncertainty quantification for dynamic flux balance analysis using non-smooth polynomial chaos expansions

**DOI:** 10.1371/journal.pcbi.1007308

**Published:** 2019-08-30

**Authors:** Joel A. Paulson, Marc Martin-Casas, Ali Mesbah

**Affiliations:** Department of Chemical and Biomolecular Engineering, University of California, Berkeley, Berkeley, California, United States of America; University of Connecticut School of Medicine, UNITED STATES

## Abstract

We present a novel surrogate modeling method that can be used to accelerate the solution of uncertainty quantification (UQ) problems arising in nonlinear and non-smooth models of biological systems. In particular, we focus on dynamic flux balance analysis (DFBA) models that couple intracellular fluxes, found from the solution of a constrained metabolic network model of the cellular metabolism, to the time-varying nature of the extracellular substrate and product concentrations. DFBA models are generally computationally expensive and present unique challenges to UQ, as they entail dynamic simulations with discrete events that correspond to switches in the active set of the solution of the constrained intracellular model. The proposed non-smooth polynomial chaos expansion (nsPCE) method is an extension of traditional PCE that can effectively capture singularities in the DFBA model response due to the occurrence of these discrete events. The key idea in nsPCE is to use a model of the singularity time to partition the parameter space into two elements on which the model response behaves smoothly. Separate PCE models are then fit in both elements using a basis-adaptive sparse regression approach that is known to scale well with respect to the number of uncertain parameters. We demonstrate the effectiveness of nsPCE on a DFBA model of an *E. coli* monoculture that consists of 1075 reactions and 761 metabolites. We first illustrate how traditional PCE is unable to handle problems of this level of complexity. We demonstrate that over 800-fold savings in computational cost of uncertainty propagation and Bayesian estimation of parameters in the substrate uptake kinetics can be achieved by using the nsPCE surrogates in place of the full DFBA model simulations. We then investigate the scalability of the nsPCE method by utilizing it for global sensitivity analysis and maximum a posteriori estimation in a synthetic metabolic network problem with a larger number of parameters related to both intracellular and extracellular quantities.

## Author summary

Construction and validation of mathematical models in biological systems involving genome-scale biomolecular networks is a challenging problem. This article presents a novel surrogate modeling method that can accelerate parameter inference from experimental data and the quantification of uncertainty in the predictions of complex dynamic biological models, with a particular emphasis on nonlinear models with non-smooth behavior. The method is applied to infer extracellular kinetic parameters in a batch fermentation reactor consisting of diauxic growth of *E. coli* on a glucose/xylose mixed media as well as a larger synthetic metabolic network problem. The proposed approach enables rigorous quantification of parameter uncertainty to determine whether or not available data is sufficient for estimation of all unknown model parameters.

## Introduction

The utility of mathematical modeling in biology is on the rise due to computational advancements and the increasing availability of data provided by high-throughput experimental techniques [1]. Flux balance analysis (FBA) is widely used for modeling cellular metabolism in a large range of metabolic and biochemical engineering problems [2, 3]. Given a constrained metabolic network, FBA assumes the intracellular fluxes are regulated by the cell to optimize a predefined cellular objective function (e.g., maximizing the biomass growth rate [4]) subject to mass balances of the intracellular metabolites and other feasibility constraints (e.g., bounds on the substrate uptake and product secretion rates). However, FBA only identifies metabolic flux distributions at steady-state and, thus, provides no information on metabolite concentrations or the dynamic behavior of the fluxes. A dynamic extension to FBA, commonly referred to as dynamic FBA (DFBA), was originally developed in [5] and has been subsequently applied in several applications [6–9]. In DFBA models, the intracellular fluxes are given by the solution of a FBA model, which is coupled to a set of dynamic equations that describes the time-varying nature of the extracellular substrate and product concentrations as a function of the extracellular environment [10]. The key assumption in DFBA is that the intracellular fluxes equilibrate instantaneously. This “quasi steady-state” assumption is valid as long as the intracellular dynamics are significantly faster than the extracellular dynamics.

Generally, the prediction of the behavior of biological systems such as those described by DFBA models can be subject to various sources of uncertainty including unknown model parameters, unknown model structure, and experimental uncertainty such as measurement error [11]. Accurate quantification of these uncertainties, as well as their impact on the quality of model predictions, is vital when applying these models in decision-support or optimization tasks such as parameter estimation or optimal experiment design. The task of uncertainty quantification (UQ) can be divided into two major problems: *forward* uncertainty propagation and *inverse* uncertainty estimation. The forward problem focuses on propagating all uncertainties through the model to predict the overall uncertainty in the outputs, whereas the inverse problem aims to calibrate the model with experimental data [12–14]. However, the most commonly used UQ methods are intractable for expensive-to-evaluate computational models [15], which has severely limited their application to DFBA models. An overview of the various challenges in DFBA simulations can be found in [16].

Surrogate modeling techniques are being increasingly adopted to enable complex UQ analyses that would otherwise be impossible. Of the available surrogate modeling approaches, polynomial chaos expansions (PCEs) are one of the most commonly used methods for UQ, which have been shown to yield accurate representations of model outputs using limited computational resources in various engineering systems [17–20] as well as biological systems [21–23]. However, an important underlying assumption in PCE is that the model response is a smooth function of the uncertain parameters such that the response can be accurately approximated by a collection of polynomial functions. For non-smooth models, PCE has been shown to either converge very slowly or even fail to converge altogether depending on the type of non-smoothness [24, 25]. This is a critical challenge in DFBA models because they are known to become singular (i.e., lose differentiability) at certain time points due to the underlying quasi steady-state assumption [10, 26, 27], meaning that even state-of-the-art PCE methods are not directly applicable to DFBA models.

In this work, we propose an extension to PCE, referred to as non-smooth PCE (nsPCE), that can adequately capture the non-smooth behavior exhibited by DFBA models. The underlying concept behind the proposed nsPCE framework is that the time of occurrence of any singularity in a DFBA model is a smooth function of the parameters, which can be effectively modeled with a PCE. Thus, for any given time of interest, the PCE model of the singularity time can be used to partition the parameter space into two non-overlapping regions (or elements) that represent the collection of parameters for which the singularity has and has not occurred. Separate PCEs can then be constructed over each of these elements, leading to a piecewise polynomial approximation of the overall model response. We adopt a non-intrusive, regression-based approach for PCE construction from a limited number of expensive DFBA simulations. In particular, we take advantage of state-of-the-art sparse regression methods to systematically locate the terms that have the greatest impact on the model response out of a very large candidate set of terms. By exploiting sparsity, we can mitigate the curse-of-dimensionality that can plague traditional PCE, allowing the application of the proposed nsPCE approach to problems with reasonably large number of uncertain parameters.

To demonstrate the effectiveness of the nsPCE method, it is applied to accelerate Bayesian estimation of parameters in the substrate uptake kinetic expressions of diauxic growth of a batch monoculture of *Escherichia coli* on a glucose and xylose mixed media. The metabolic network reconstruction used for *E. coli* is iJ904, which is a genome-scale model that contains 1075 reactions and 761 metabolites [28]. Parameter estimation is performed using measurements of the concentrations of extracellular metabolites and biomass that are taken at certain time points throughout the batch. We selected this particular system due to the fact that reported parameter estimates were determined from experimental data using a trial-and-error procedure [8]. This was likely due to the computational complexity of the genome-scale DFBA model in conjunction with the limited data set that may not enable unique estimation of parameters. In addition, we demonstrate how nsPCE can be applied to vastly speedup forward UQ analyses including global sensitivity analysis and estimation of the probability distribution of the model response. To demonstrate the scalability of nsPCE, it is used for maximum a posteriori parameter estimation in a synthetic metabolic network problem with twenty unknown parameters related to quantities in both the intracellular reaction network and the extracellular environment. The codes that implement the proposed nsPCE method for generic DFBA models are provided at the repository [29].

## Methods

### Dynamic flux balance analysis models

We focus on modeling a microbial cultivation process using dynamic flux balance analysis (DFBA), in which the bioreactor is viewed as a combination of the fluid medium (extracellular environment) and the microorganisms (intracellular environment). Cell walls act as physical boundaries between these two phases, through which certain chemical metabolites are exchanged. The DFBA model can be mathematically formulated as [26]

$$\begin{array}{c} \dot{s}\left(t\right)=f\left(t,s\left(t\right),v\left(s\left(t\right)\right)\right),\;\;s\left(t_{0}\right)=s_{0}, \end{array}$$ (1)

with **v**(**s**(*t*)) being an element of the solution set of the flux balance model

$$\begin{array}{cc} v\left(s\right)\in \underset{v}{\text{argmax}}\;\; & h(v,s)\\ \text{subject}\;\text{to:}\;\; & Av=0,\\ & v^{\text{LB}}\left(s\right)\leq v\leq v^{\text{UB}}\left(s\right), \end{array}$$ (2)

where **s** denotes the state variables describing the extracellular environment (e.g., concentrations of substrates, biomass, and products) with time derivative $\dot{s}$ and initial conditions **s**_0_; **v** denotes the metabolic fluxes that include both intracellular fluxes and exchange rates; **A** is the stoichiometric matrix of the metabolic network; and **v**^LB^(**s**) and **v**^UB^(**s**) are the lower and upper bounds on the fluxes, respectively, which are functions of the extracellular concentrations. The vector function **f**, specified by the set of mass balances in the extracellular medium, defines the rate of change of each component of **s** and must be integrated to determine the time evolution of extracellular concentrations. The scalar function *h* is the cellular objective that is maximized by the cells. Whenever more than one microbial species are present in the culture, then multiple flux balance models of the form (2) must be incorporated into (1) [10].

DFBA models can be classified as ordinary differential equations with embedded optimization wherein the lower-level FBA optimization can either be a linear or nonlinear program [30]. A variety of methods have been developed for integrating DFBA models, which are summarized in S1 Text. We focus on the direct approach for integrating DFBA models in this work due to its ability to ensure accurate solutions through the use of error-controlled integration schemes. Another advantage of the direct approach is that a unique solution set to the FBA (2) can be obtained using lexicographic optimization [10, 27], which may help overcome numerical challenges that can occur when using alternative DFBA simulators (e.g., see [31, Chapter 3]). Since the direct approach requires continuous monitoring and identification of any active set changes in (2), it constitutes a dynamic simulation with discrete events (i.e., a hybrid system). In the next section, we present the proposed nsPCE method that is capable of directly accounting for the hybrid nature of DFBA models.

### Polynomial chaos expansions

#### Theoretical background

We consider a DFBA model with a set of *M* input parameters that are denoted by ***x*** = (*x*_1_, …, *x*_*M*_). These parameters can appear in the initial conditions **s**_0_, rate of change function **f**, cellular objective *h*, and/or the flux limits **v**^LB^, **v**^UB^. We look to develop a computationally cheap-to-evaluate representation of some output of the DFBA model referred to as the *model response*. The model response y=M(x) can be any chosen function of the states or fluxes that appear in (1) and (2) including, for example, metabolite concentrations, growth rate, or time-to-consumption of any metabolite. The model response function $M:R^{M}\to R$ need not be known analytically, and can be approximated using a finite number of model evaluations. We focus on the scalar response case *y* for notational simplicity. However, the developed procedure can be easily applied separately to each component of a vector of responses ***y***.

Unless the parameters ***x*** are perfectly known, they must be treated as uncertain. Parameter uncertainty can generally be represented by a random vector ***X*** with some known probability density function (PDF). In this case, the model response also becomes a random variable with some unknown PDF that is implicitly defined by

$$\begin{array}{c} Y=M\left(X\right),\;\;\;\;X∼f_{X}, \end{array}$$ (3)

where ∼ denotes “distributed as” and *f*_***X***_ denotes the PDF of uncertain parameters. Determining the distribution *f*_***Y***_ (or its statistical moments) of the model response represents the forward UQ problem that can be tackled in various ways, the majority of which require extensive sampling that is not feasible whenever M is a computationally expensive model. The polynomial chaos expansion (PCE) method addresses this problem by constructing a *surrogate model* that accurately approximates M, but is significantly cheaper to evaluate. The PCE surrogate model can also be straightforwardly applied to other UQ tasks, as discussed later in the Results section. Provided that *Y* has finite variance, it can be represented with a PCE as follows [17]

$$\begin{array}{c} Y=M\left(X\right)=\sum_{\alpha \in N^{M}}a_{\alpha }\Psi_{\alpha }\left(X\right), \end{array}$$ (4)

where $a_{\alpha }\in R$ are coefficients of the expansion, $\Psi_{\alpha }:R^{M}\to R$ are multivariate polynomials, ***α*** = (*α*_1_, …, *α*_*M*_) is a multi-index that identifies the degree of the multivariate polynomials in each of the input parameters *X*_*i*_, and N={1,2,…} is the set of positive integers. The polynomial basis functions are required to be orthonormal with respect to the parameter distribution, such that they satisfy

$$\begin{array}{c} E\left\{\Psi_{\alpha }\left(X\right)\Psi_{\beta }\left(X\right)\right\}=\int_{S}\Psi_{\alpha }\left(x\right)\Psi_{\beta }\left(x\right)f_{X}\left(x\right)dx=\delta_{\alpha \beta },\;\;\;\;\forall \alpha ,\beta \in N^{M}, \end{array}$$ (5)

where *S* is the support of the distribution of ***X*** and *δ*_***αβ***_ is the Kronecker delta that is 1 whenever ***α*** = ***β*** and 0 otherwise. For computational purposes, the series (4) must be truncated after a finite number of *P* terms, which yields the following approximation

$$\begin{array}{c} Y^{PCE}=M^{PCE}\left(X\right)=\sum_{\alpha \in A}a_{\alpha }\Psi_{\alpha }\left(X\right)=a^{⊤}\Psi \left(X\right), \end{array}$$ (6)

where A is a finite set of multi-indices with cardinality equal to *P*, $a\in R^{P}$ is a vector of the coefficients, and $\Psi :R^{M}\to R^{P}$ is a vector containing all polynomial basis functions. The expansion coefficients are defined to be those that minimize the mean-square error (MSE) between the exact representation (4) and the truncated PCE (6)

$$\begin{array}{c} a=\underset{\tilde{a}\in R^{P}}{\text{argmin}}\;E\{{\left(M\left(X\right)-{\tilde{a}}^{⊤}\Psi \left(X\right)\right)}^{2}\}=E\left\{M\left(X\right)\Psi \left(X\right)\right\}. \end{array}$$ (7)

The right-hand side of this expression represents the analytic solution to the MSE optimization problem and directly follows from the Hilbert projection theorem [32].

The expressions in (5) and (7) involve multivariate integration over complicated nonlinear functions. As such, the construction of the polynomial basis and computation of the expansion coefficients are usually carried out numerically in practice, which leads to additional sources of error. The choice of A also plays an important role in PCE performance because A directly controls the number of coefficients that must be estimated. Larger *P* values require more computational effort and are more susceptible to numerical sources of error. An overview of state-of-the-art methods for addressing these challenges is provided next.

#### Orthonormal basis construction

The complexity of determining the polynomials ${\left\{\Psi_{\alpha }\left(X\right)\right\}}_{\alpha \in A}$ depends fully on the structure of the PDF *f*_***X***_. Whenever the uncertain parameters are statistically independent, then (5) reduces to the tensor product of *M* univariate polynomials that are orthonormal with respect to each marginal density $f_{X_{i}}$. These polynomials have been analytically derived for many common PDFs [17], and can be found numerically for generic PDFs using algorithms in terms of the three-term recurrence relationship for orthogonal polynomials [33]. There are two main approaches for handling the more general case that ***X*** has statistically dependent (or correlated) elements. The first approach involves transforming the generic random vector ***X*** into a standard random vector ***Z*** for which it is simpler to build the polynomial basis functions [34]. Any *isoprobabilistic transformation* that preserves the PDFs of these random vectors can be utilized, though the most commonly used is the Rosenblatt transformation [35]. The second approach involves applying a more sophisticated numerical procedure that is able to impose the conditions in (5) simultaneously in *M* dimensions. This includes the Gram-Schmidt process [36] as well as the modified Cholesky decomposition of the Gram moment matrix [37, 38].

#### Sparse truncation and regression

We denote the approximate PCE with numerically estimated coefficients $\hat{a}$ as follows

$$\begin{array}{c} {\hat{Y}}^{PCE}={\hat{M}}^{PCE}\left(X\right)={\hat{a}}^{⊤}\Psi \left(X\right). \end{array}$$ (8)

A variety of methods have been proposed for estimating the coefficients that can be broadly categorized as *intrusive* (e.g., Galerkin projection [12]) or *non-intrusive* (e.g., pseudo-spectral projection [39] or regression [15]). Here, we focus exclusively on non-intrusive methods. The phrase “non-intrusive” implies that coefficient estimates are obtained over a finite set of parameter realizations $X=\{x^{\left(1\right)},\ldots ,x^{\left(N\right)}\}$, referred to as the experimental design (ED). These samples can be chosen in various ways including Monte Carlo sampling, quasi-random samples derived from Sobol or Halton sequences, or sparse grids to name a few [40]. The computational model is then evaluated at every point in the ED, i.e., $Y=\{y^{\left(1\right)},\ldots ,y^{\left(N\right)}\}$ with $y^{\left(i\right)}=M\left(x^{\left(i\right)}\right)$ for all *i* = 1, …, *N*. As such, non-intrusive approaches are “black-box” in the sense that they can be applied to any function, even when this function is not explicitly known, and do not require any modification to the deterministic solver.

We will focus on regression methods due to their flexibility when it comes to enforcing sparsity. In the regression approach, coefficients $\hat{a}$ are defined as those that minimize the least-square residual of the polynomial approximation over the ED X

$$\begin{array}{c} \hat{a}=\underset{\tilde{a}\in R^{P}}{\text{argmin}}\;\frac{1}{N}\sum_{i=1}^{N}{\left(M\left(x^{\left(i\right)}\right)-{\tilde{a}}^{⊤}\Psi \left(x^{\left(i\right)}\right)\right)}^{2}={\left(A^{⊤}A\right)}^{-1}A^{⊤}Y, \end{array}$$ (9)

where $A\in R^{N\times P}$ is the model matrix that contains the values of all polynomial basis functions evaluated at all ED points. The solution of (9) requires a minimum number of sample points *N* ≥ *P* to ensure a unique solution exists. Since every sample requires an expensive DFBA simulation here, the truncation scheme plays a central role in reducing the complexity of surrogate model construction. The total degree method is the most commonly used approach for specifying A, which looks to keep all polynomials up to a specified order *p* in the series. For total degree truncation, the set of multi-indices is defined as $A=\{\alpha \in N^{M}:\|\alpha {\|}_{1}\leq p\}$, where ‖***α***‖_1_ = *α*_1_ + ⋯ + *α*_*M*_ and $P=\frac{(M+p)!}{M!p!}$. Due to the sharp increase in *P* as the polynomial order increases, the total degree truncation scheme can quickly lead to a prohibitive number of model evaluations, especially in high dimensions. This issue is often termed the *curse-of-dimensionality*, which is known to considerably limit standard PCE methods.

We look to take advantage of two approaches for overcoming the curse-of-dimensionality limitation. The first approach involves replacing the total order truncation with the so-called *hyperbolic* truncation scheme, which is defined as

$$\begin{array}{c} A^{M,p,q}=\{\alpha \in N^{M}{:\|\alpha \|}_{q}\leq {p\},\;\;\;\;\|\alpha \|}_{q}={\left(\sum_{i=1}^{M}\alpha_{i}^{q}\right)}^{1/q}, \end{array}$$ (10)

where 0 < *q* ≤ 1. Lower values for *q* limit the number of high-order interaction terms considered, which directly lead to sparser solutions. The second approach looks to further sparsify the solution, without sacrificing potentially important interaction terms, by including a regularization term of the form $\lambda \|\tilde{a}{\|}_{1}$ with λ ≥ 0 in the least-squares problem (9). This regularization term is known to force the minimization to favor low-rank solutions and ensures the existence of a unique solution even when *N* < *P*.

The key challenge with regularization is a proper choice of λ, which indirectly specifies the number of non-zero coefficients included in the expansion. In this work, we use the hybrid least angle regression (LAR) method to solve the regularized version of (9). LAR is an efficient procedure for variable selection, which aims to select the predictors (i.e., polynomials Ψ_***α***_) that have the greatest impact on the model response among a potentially large set of candidates [41]. Hybrid LAR is a variant of the original LAR that uses a modified cross-validation scheme to estimate the approximation error [19]. This modification relies on only a single call to the LAR procedure, which provides significant savings in computational cost when compared to the original method. The relative MSE (RMSE), which is defined as *ε* = MSE/Var{*Y*}, is the natural choice of the approximation error in PCE and can be robustly estimated by the leave-one-out (LOO) cross-validation error *ε*_*LOO*_. Not only can *ε*_*LOO*_ be calculated analytically for PCE models [42], but it is known to be much less sensitive to overfitting than the empirical estimator [43].

Provided a sensible sampling strategy has been chosen, the remaining parameters that must be selected are related to truncation *p* and *q* and the ED size *N*. We use a systematic procedure for selecting these parameters to achieve a target error level *ε*_*target*_. As discussed in [19], a *basis-adaptive* strategy can help overcome potential limitations of an *a priori* fixed truncation set A by letting the maximum degree be driven directly from the data. The basic idea is to start with small values for *p* and *q*, estimate the coefficients using hybrid LAR, and calculate *ε*_*LOO*_. These steps are repeated for incremented values of *p* and *q*, and the algorithm returns the PCE model with the lowest error. Early stop criteria can easily be introduced to avoid an excessive number of iterations. However, when dealing with computationally expensive models, the number of model evaluations *N* dominates the cost of construction of the surrogate model. We therefore propose an iterative “greedy” approach for constructing the ED to ensure that *N* can be kept as small as possible. This sequential ED strategy can be summarized as

1. Initialize the current ED with a relatively small number of samples *N*_*init*_.
2. Train a sparse basis-adaptive PCE using the current ED and calculate *ε*_*LOO*_.
3. If *ε*_*LOO*_ < *ε*_*target*_, stop the algorithm and return current PCE. Otherwise, enrich the current ED with *N*_*add*_ more samples and return to Step 2.

Note that any method can be used in the training step of this algorithm. Thus, in the proposed nsPCE method, the desired accuracy level is the key parameter that must chosen by the user.

### The nsPCE surrogate modeling method

The PCE method is guaranteed to converge as both the number of model evaluations *N* and number of terms in the expansion *P* increase; however, the rate of convergence can be very slow whenever M exhibits any singularities [24]. This is a primary challenge in DFBA models since they can lose differentiability when a switch in the active set of the FBA problem (2) occurs. Inspired by [25], we look to take advantage of the following *multi-element* representation of PCE as it is capable of capturing non-smooth behavior

$$\begin{array}{c} Y=M\left(X\right)=\sum_{k=1}^{N_{e}}\sum_{\alpha \in N^{M}}a_{k,\alpha }\Psi_{k,\alpha }\left(X\right)I_{S_{k}}\left(X\right), \end{array}$$ (11)

where *N*_*e*_ denotes the number of elements; *S*_*k*_, *a*_*k*,***α***_, and Ψ_*k*,***α***_ denote the local support, coefficient, and orthogonal polynomials in element *k*, respectively; $S={⋃}_{k=1}^{N_{e}}S_{k}$; and $I_{S_{k}}\left(X\right)$ are indicator random variables defined by

$$\begin{array}{c} I_{S_{k}}\left(X\right)=\{\begin{array}{c} 1\;\;\text{if}\;X\in S_{k}\\ 0\;\;\text{otherwise}. \end{array}\;\;k=1,\ldots ,N_{e} \end{array}$$ (12)

The indicator random variables can be used to define the following conditional random variables $X_{k}=X|\left(I_{S_{k}}\left(X\right)=1\right)$ with PDF

$$\begin{array}{c} f_{X_{k}}\left(x_{k}\right)=\frac{f_{X}\left(x_{k}\right)}{\text{Pr}(I_{S_{k}}\left(X\right)=1)}=\frac{f_{X}\left(x_{k}\right)}{\int_{S_{k}}f_{X}\left(x\right)dx}. \end{array}$$ (13)

The local polynomials in (11) are orthogonal with respect to ***X***_*k*_ while the coefficients are similarly defined as in (7) but now in terms of ***X***_*k*_. This implies that the same strategies discussed above for building the polynomials, estimating the coefficients using regularized least squares, truncating the expansion, and sequentially populating the ED can be utilized locally within each element.

The remaining unanswered question is how to design the elements ${\left\{S_{k}\right\}}_{k=1}^{N_{e}}$ to limit the growth in the number of model evaluations since *N* will scale approximately linearly with *N*_*e*_. The best decomposition should ensure that the model response behaves smoothly in every element. The proposed nsPCE method decomposes the support into two elements *S*_1_ and *S*_2_ that denote, respectively, the set of parameters for which the singularity has not and has occurred. This idea is best illustrated through a simple example. Consider the following non-smooth ODE system $\dot{y}=-x$ if *y* > 0 and $\dot{y}=0$ otherwise with initial condition *y*_0_ > 0, whose solution is given by

$$\begin{array}{c} y\left(t,x\right)=\{\begin{array}{cc} y_{0}-tx, & \text{if}\;y_{0}>tx,\\ 0, & \text{otherwise}. \end{array} \end{array}$$ (14)

This function is not differentiable at the time point *t*_*s*_(*x*) = *y*_0_/*x*, which can be thought of as the “singularity manifold” in the parameter support space, i.e., *t*_*s*_ is the boundary function that separates *S*_1_ and *S*_2_. At any given time of interest *t*, the two elements can be defined in terms of *t*_*s*_(*x*) as follows

$$\begin{array}{c} S_{1}\left(t\right)=\left\{x\in S:t_{s}\left(x\right)>t\right\},\;\;\;\;S_{2}\left(t\right)=\left\{x\in S:t_{s}\left(x\right)\leq t\right\}. \end{array}$$ (15)

Let us briefly analyze the behavior of these elements. The elements are continuous functions of time, meaning that every time of interest *t* requires a different decomposition. Whenever *t* is outside of the support of *t*_*s*_(*X*), then one of these sets is empty and we revert back to traditional PCE that covers the full support *S*. In light of this, we can easily generalize the idea to the case of multiple *n*_*s*_ > 1 sequential singularities as long as the random variables ${\left\{t_{s_{i}}\left(X\right)\right\}}_{i=1}^{n_{s}}$ do not have overlapping supports. When multiple non-overlapping singularities are present, we must simply find the support in which *t* lies and define the two elements using that corresponding boundary function. The case of overlapping supports is more challenging due to the fact that more elements would need to be created based on the intersection of *S*_1_ and *S*_2_ for all active singularities.

For the simple scalar example in (14), we can analytically derive the boundary function; however, this is not generally possible in DFBA models. Based on the observation that the singularity boundary depends smoothly on the parameters, we instead propose to construct a sparse PCE model to approximate the boundary in multiple dimensions, i.e., $t_{s}\approx {\hat{t}}_{s}^{PCE}$. The nsPCE method thus creates a surrogate model with the following structure for any ***x*** ∈ *S*

$$\begin{array}{c} {\hat{M}}^{nsPCE}\left(x\right)=\sum_{k=1}^{2}{\hat{a}}_{k}^{⊤}\Psi_{k}\left(x\right)I_{S_{k}}\left(x\right)=\{\begin{array}{c} {\hat{a}}_{1}^{⊤}\Psi_{1}\left(x\right),\;\;\text{if}\;x\in S_{1},\\ {\hat{a}}_{2}^{⊤}\Psi_{2}\left(x\right),\;\;\text{if}\;x\in S_{2}, \end{array} \end{array}$$ (16)

where the coefficients ${\hat{a}}_{k}$ are estimated from the sparse regression problem

$$\begin{array}{c} {\hat{a}}_{k}=\underset{{\tilde{a}}_{k}}{\text{argmin}}\;\frac{1}{N_{k}}\|Y_{k}-A_{k}{\tilde{a}}_{k}{\|}_{2}^{2}+\lambda_{k}{\left\|{\tilde{a}}_{k}\right\|}_{1},\;\;\;\;k\in \left\{1,2\right\} \end{array}$$ (17)

based on the local ED $X_{k}=\left\{x_{k}^{\left(1\right)},\ldots ,x_{k}^{\left(N_{k}\right)}\right\}$ and $Y_{k}=\left\{y_{k}^{\left(1\right)},\ldots ,y_{k}^{\left(N_{k}\right)}\right\}$ in terms of *N*_*k*_ samples. Notice that the full DFBA model must be integrated when constructing ${\hat{t}}_{s}^{PCE}$. Instead of discarding this information, it can be reused by storing the list of state and time points generated when integrating the DFBA model and then interpolating these points when calculating the model response function. Thus, we can use this approach to initialize the ED X, model response data Y, and singularity time data $T_{s}$. Using $T_{s}$ along with the set definitions in (15), we can easily partition X and Y into the required local EDs. The sequential ED strategy is then applied in each element to ensure that the target error is achieved.

**Fig 1:**
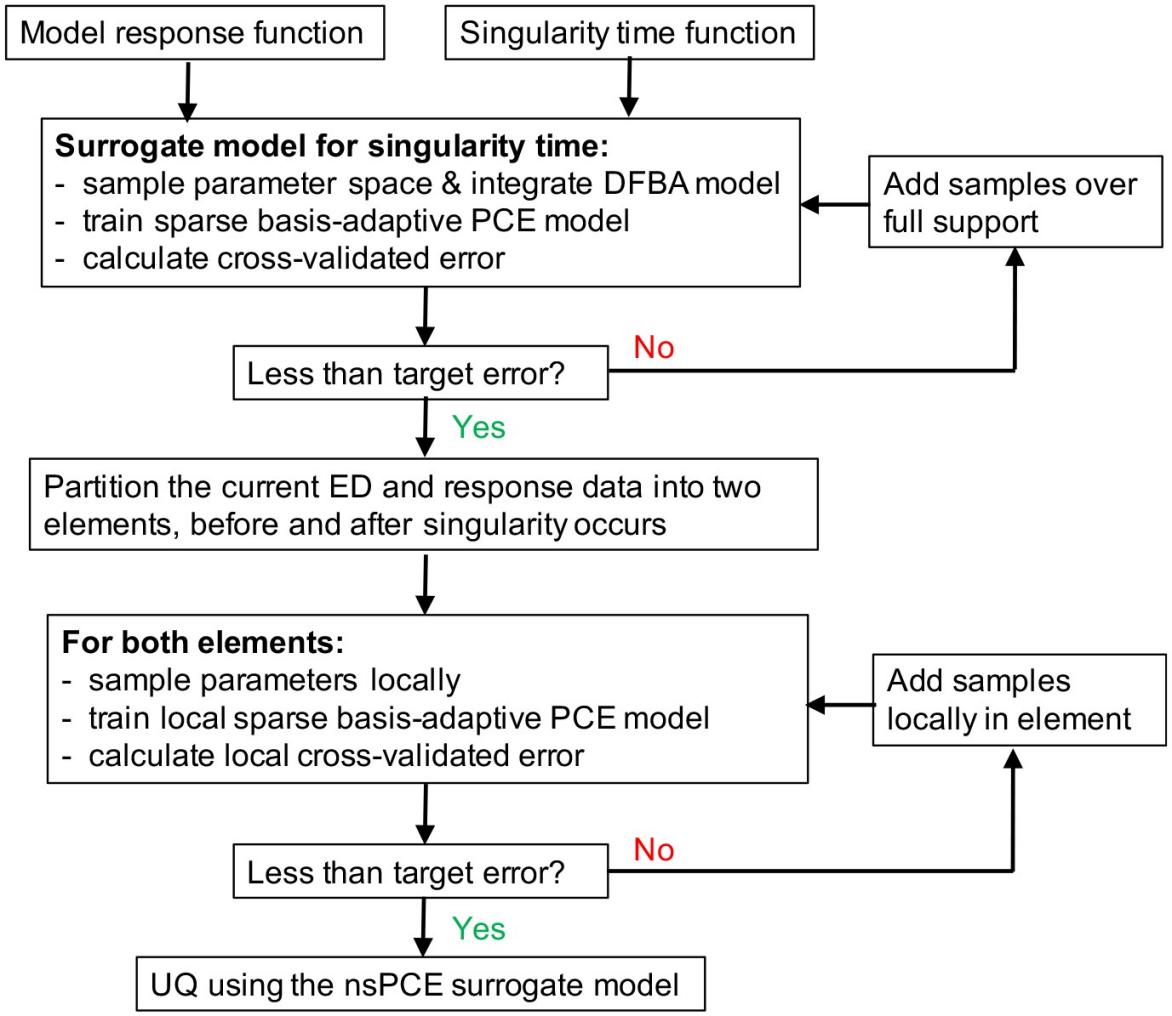
Flowchart for the proposed nsPCE surrogate modeling method. The model response function can be freely chosen by the user. The singularity time function should be specified implicitly as a function of the DFBA model states. This function can be identified by simulating the DFBA model with nominal parameters and locating at which time points a switch in the active set of the FBA solution occurs. The PCE coefficients are fit using the basis-adaptive version of the hybrid LAR method, while the ED is sequentially enriched to ensure that the target accuracy level is achieved.

A flowchart that summarizes the main steps of the nsPCE method is shown in Fig 1. By evaluating the nsPCE surrogate in (16), which is much cheaper to evaluate than the full model M, on a collection of Monte Carlo samples of the parameters, we can directly approximate statistical properties of *Y* including moments, parametric sensitivities, or even its full distribution.

### Numerical implementation

The complete set of Matlab scripts that implement the nsPCE method is available at [29]. All of the modifiable parameters in the algorithm are defined in the “User inputs” section of the main_pce.m script, which automatically executes the steps summarized in Fig 1. It is important to note that the scripts require the installation of two additional packages that integrate the DFBA model and construct sparse PCE models. The nsPCE scripts are written to be compatible with readily available DFBA and PCE toolboxes to provide flexibility. The simulation of DFBA models can be done with any non-smooth integration code including COBRA [44], ORCA [45], and DFBAlab [10]. All files needed by the DFBA integrator should be placed in the dfba_model folder. We opt for DFBAlab in this work due to certain numerical advantages that it exhibits over the available alternatives (see [27, 31] for more details). The sparse PCE operations are carried out using UQLab [43], which implements the hybrid LAR method as well as the required calculations to determine the cross-validation error *ε*_*LOO*_. The syntax in main_pce.m is heavily based on UQLab. Hence, some modifications to the source code may be needed to perform the same operations with other toolboxes.

## Results

We present two separate case studies in this section. The first case study explores Bayesian estimation of six parameters related to the substrate uptake kinetics in a computationally expensive DFBA model of *E. coli* with a genome-scale metabolic network. The goal of the first case study is to demonstrate advantages of the proposed nsPCE method over alternatives as well as its application to a realistic problem that has been previously studied in the literature. The second case study focuses on maximum a posteriori estimation in a synthetic DFBA problem with a relatively large number of parameters, i.e., twenty uncertain parameters appearing in a variety of intracellular and extracellular quantities. The goal of the second case study is to provide preliminary evidence of the scalability of nsPCE as well as the fact that the method is applicable to a wide-variety of UQ applications.

### Case study 1: Batch fermentation of *E. coli* monoculture

This case study is based on a DFBA model of a batch fermentation reactor consisting of an *E. coli* monoculture, which has been investigated for the production of valuable chemicals such as ethanol. Here, we focus on the initial phase of batch operation of the *E. coli* fermentation reactor under aerobic growth in a glucose and xylose mixed media [8]. No ethanol production is observed under aerobic conditions (i.e., this phase is mainly used to increase the biomass), such that the concentration of ethanol can be omitted from the dynamics. This case study is commonly used as a benchmark for comparing DFBA solvers (see, e.g., [16, 27, 31]), as it exhibits stiff dynamics and multiple singularities.

The dynamic mass balance equations of the form (1) for the extracelluar environment can be summarized as follows

$$\begin{array}{c} \begin{array}{cc} \dot{b}\left(t\right) & =\mu (t)b(t),\\ \dot{g}\left(t\right) & =-u_{g}\left(t\right)b\left(t\right),\\ \dot{z}\left(t\right) & =-u_{z}\left(t\right)b\left(t\right), \end{array} \end{array}$$ (18)

where *b*(*t*), *g*(*t*), and *z*(*t*) denote the biomass, glucose, and xlyose concentrations at time *t*, respectively. The uptake kinetics for glucose, xylose, and oxygen are given by Michaelis-Menten kinetics

$$\begin{array}{c} \begin{array}{cc} u_{g}\left(t\right) & =u_{g,max}\frac{g(t)}{K_{g}+g\left(t\right)},\\ u_{z}\left(t\right) & =u_{z,max}\frac{z(t)}{K_{z}+z\left(t\right)}\frac{1}{1+\frac{g(t)}{K_{ig}}},\\ u_{o}\left(t\right) & =u_{o,max}\frac{o(t)}{K_{o}+o\left(t\right)}, \end{array} \end{array}$$ (19)

where parameters *u*_*g*,*max*_, *u*_*z*,*max*_, *u*_*o*,*max*_, *K*_*g*_, *K*_*z*_, *K*_*o*_, and *K*_*ig*_ correspond to the maximum substrate uptake rates, saturation constants, and inhibition constants. It is assumed that the reactor oxygen concentration, *o*(*t*), is controlled and is therefore constant. The growth rate *μ*(*t*), on the other hand, is determined from the metabolic network model of wild-type *E. coli*. The chosen metabolic network reconstruction was iJR904 [28], which contains 1075 reactions and 761 metabolites. The cells are assumed to maximize growth, implying (2) is an LP of the form

$$\begin{array}{c} \begin{array}{cc} \mu \left(t\right)=\underset{v}{\text{min}} & \;\;c^{⊤}v,\\ \text{s}.\text{t}. & \;\;Av=0,\\ & \;\;v_{g_{ext}}=u_{g}\left(t\right),\\ & \;\;v_{z_{ext}}=u_{z}\left(t\right),\\ & \;\;v_{o_{ext}}=u_{o}\left(t\right),\\ & \;\;v^{\text{LB}}\leq v\leq v^{\text{UB}}, \end{array} \end{array}$$ (20)

where **c** is a vector of weights that represent the contribution of each flux to biomass formation while $v_{g_{ext}}$, $v_{z_{ext}}$, and $v_{o_{ext}}$ are, respectively, the exchange fluxes for glucose, xylose, and oxygen (i.e., elements of the flux vector **v**). Thus, the metabolic network interacts with the extracellular environment through the exchange fluxes in (19).

The initial conditions of the batch are assumed to be fixed at 0.03 g/L of inoculum, 15.5 g/L of glucose, and 8 g/L of xylose; the oxygen concentration is kept constant at 0.24 mmol/L; and **A**, **c**, **v**^LB^, and **v**^UB^ are specified by the iJR904 model. However, the parameters in the substrate uptake rates (19) should be fit to experimental data since they cannot be easily predicted from first principles. This problem of identifying the model parameters was partially tackled in [8], where most of the parameters were fixed according to estimates provided in the literature while *u*_*z*,*max*_ and *K*_*ig*_ were adjusted by trial-and-error to match transient measurements of biomass, glucose, and xylose. The reported parameter estimates are given in S1 Table. Since *o*(*t*) is fixed, *u*_*o*,*max*_ and *K*_*o*_ can be lumped into a single parameter *u*_*o*_. These six parameters are unknown and here are modeled as a random vector whose elements are independent and uniformly distributed around ±10% of the nominal values. We selected this range to reflect a reasonable level of confidence in the reported literature values. In the following, we demonstrate how the proposed nsPCE surrogate modeling method can facilitate UQ tasks that are otherwise computationally intractable with respect to the full DFBA model.

All reported computations are performed in MATLAB R2016a on a MacBook Pro with 8 GB of RAM and a 2.6 GHz Intel i5 processor. The DFBA model is simulated using DFBAlab with default options for integration and LP optimization tolerances. CPLEX was used as the LP solver and MATLAB ode15s was used as the integrator.

#### Decomposition of parameter space

**Fig 2:**
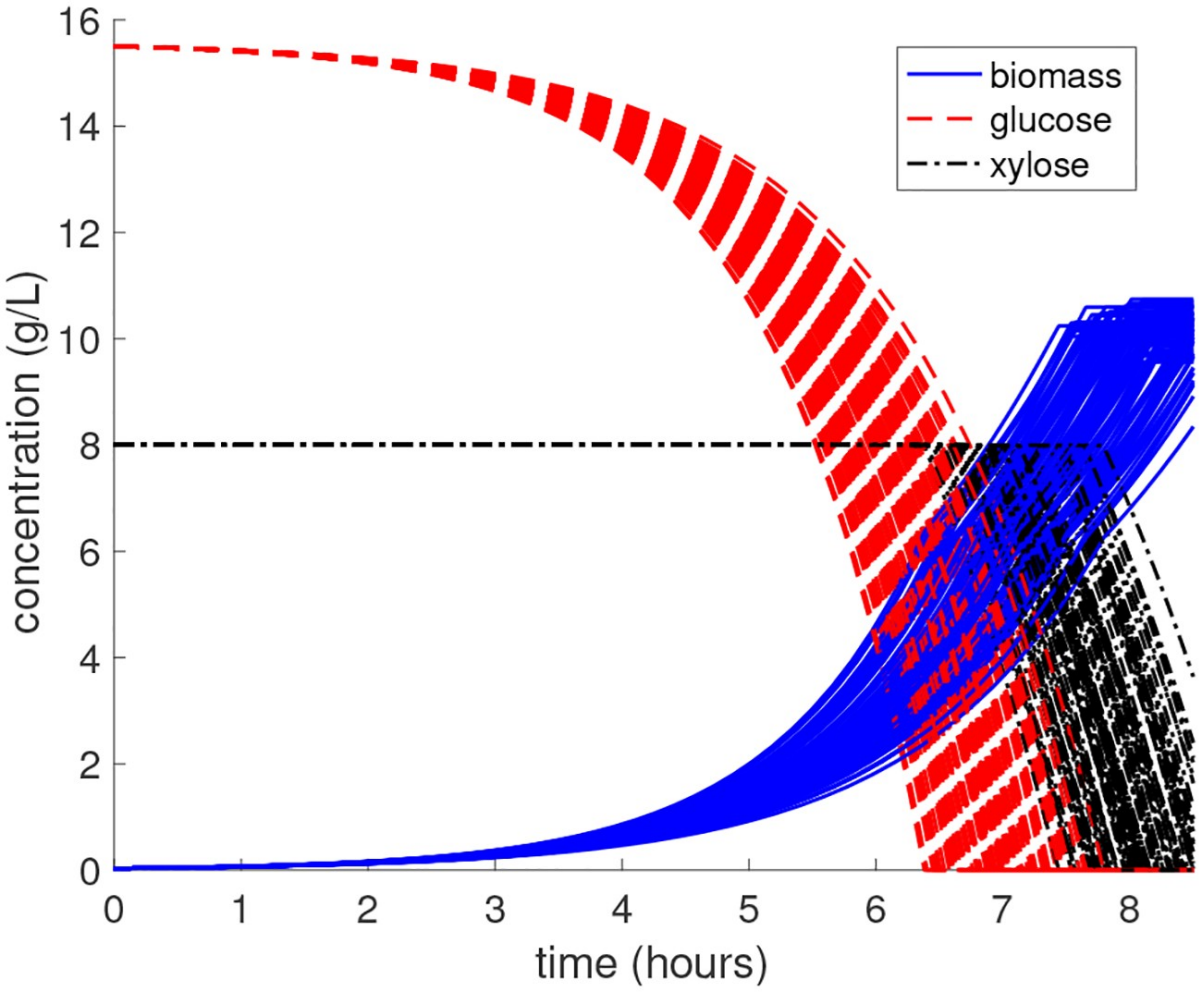
Monte Carlo simulation of *E. coli* DFBA model. The genome-scale model is integrated from 0 to 8.5 hours for 100 different parameter realizations that are independently drawn from the uniform prior density. The consumption of xylose only occurs after glucose is fully exhausted, which is a strong function of the parameters.

Before selecting the element decomposition, we must first simulate the DFBA model to locate any significant singularities. The extracellular glucose, xylose, and biomass concentration profiles are plotted in Fig 2 for one hundred randomly sampled parameter values. For a given realization of the parameter, the full simulation requires approximately 1.5 seconds of CPU time.

**Fig 3:**
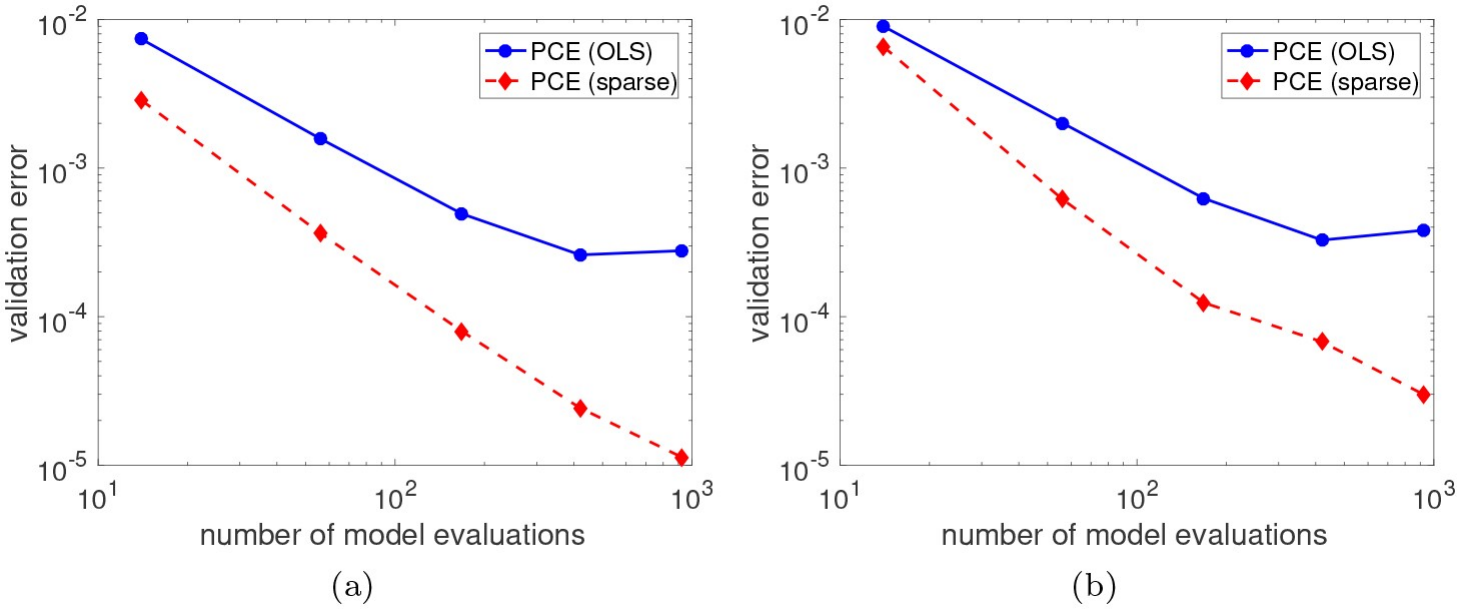
Accuracy of singularity time surrogate models. RMSE versus the number of model evaluations (i.e., size of the experimental design) used to train the PCE model for **(a)** the glucose singularity *t*_*g*_ and **(b)** the xylose singularity *t*_*z*_. The RMSE was estimated empirically from a validation set of 10,000 full DFBA simulations.

At the start of the batch, glucose is consumed preferentially over xylose. Once glucose has been depleted, the LP solution switches and xylose becomes the main carbon source. The final batch time is then specified as the time that both glucose and xylose have been fully depleted, at which point the LP becomes infeasible and the solution ceases to exist. The *E. coli* cells stop growing at this point due to the lack of a carbon source. Although physically the cells would begin to die in this situation, DFBA models cannot directly predict the cell death phase and thus we assume the biomass remains constant for simplicity. The time-to-consumption of glucose *t*_*g*_ and xylose *t*_*z*_ represent the two singularities in this problem, and clearly depend on the value of the model parameters. Since the singularity time functions cannot be derived analytically, we look to construct PCE approximations for both *t*_*g*_ and *t*_*z*_. We investigate two different fitting methods: classical *full* PCE with coefficients estimated using ordinary least squares (OLS) and *sparse* basis-adaptive PCE with coefficients estimated using hybrid LAR. The degree of the polynomials is varied from 1 to 6 in the full PCE method, where *N* = 2*P* model evaluations are used for regression with *P* denoting the size of the basis. In the sparse PCE method, the maximum degree is allowed to vary from 1 to 20, and a hyperbolic truncation scheme (10) is used with *q* = 0.75. The experimental designs (EDs) are generated using Monte Carlo (MC) sampling with a fixed random seed to ensure repeatable results. Fig 3a and 3b show the RMSE as a function of the number of model evaluations used to fit surrogate models for *t*_*g*_ and *t*_*z*_, respectively. The sparse PCE method consistently outperforms full PCE, achieving approximately an order-of-magnitude lower RMSE for all ED sizes.

**Fig 4:**
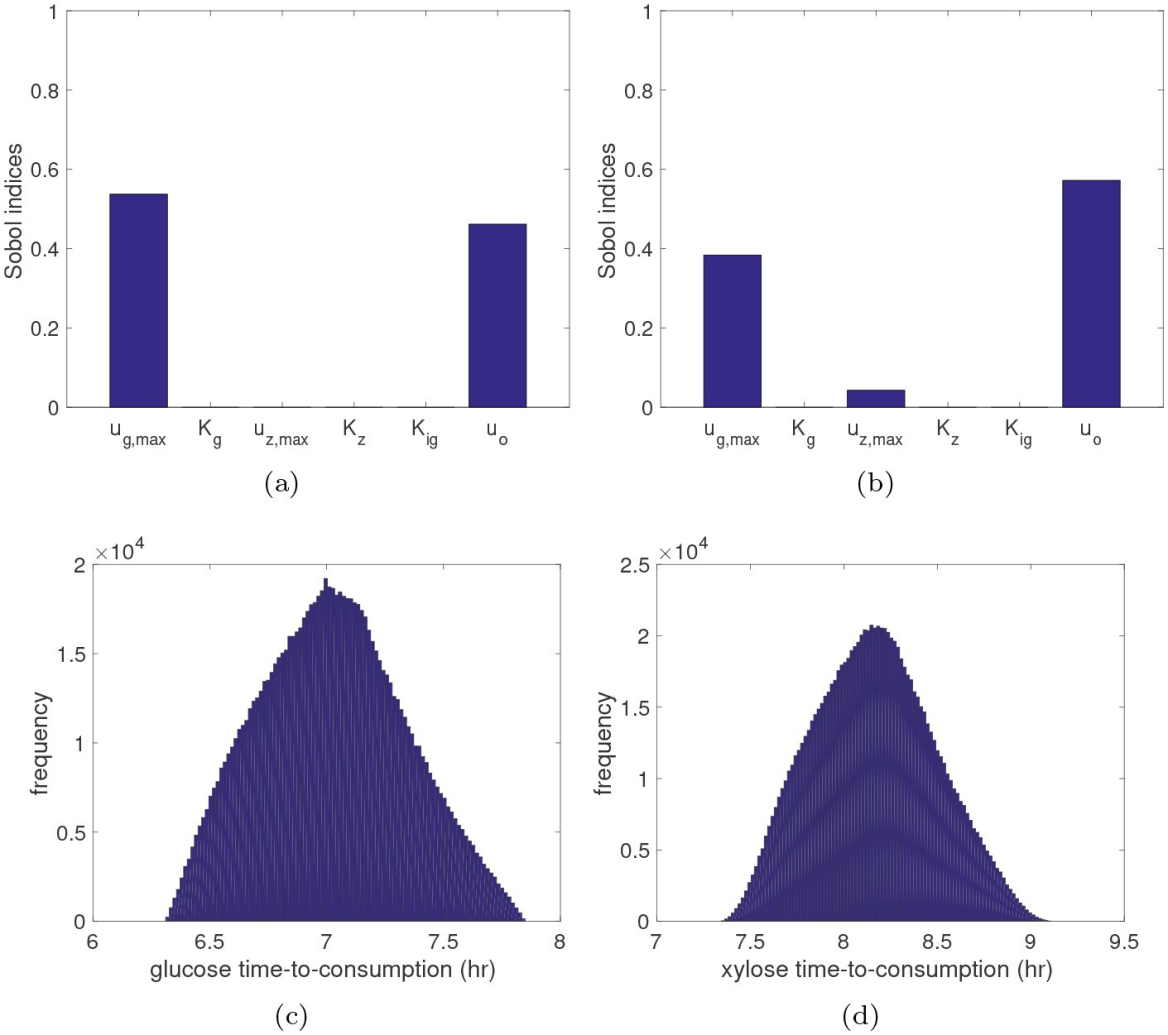
Uncertainty propagation with singularity time surrogate models. The estimated global sensitivity indices of **(a)** *t*_*g*_ and **(b)** *t*_*z*_ with respect to the uncertain parameters. The estimated PDF of **(c)** *t*_*g*_ and **(d)** *t*_*z*_ based on 1e+6 surrogate model evaluations, which only requires approximately 1 second of CPU time.

The sparse PCE surrogate models for *t*_*g*_ and *t*_*z*_ are used in the nsPCE method to build surrogates for the extracellular concentrations. Additionally, these surrogate models contain useful information on which parameters influence the consumption of different substrates. The Sobol’ indices of *t*_*g*_(***X***) and *t*_*z*_(***X***) are shown in Fig 4, which are a commonly used tool in global sensitivity analysis for ranking the parameters according to their contribution to the variance of the model response. The Sobol’ indices can be computed *analytically* from the PCE coefficients [43], which requires less than one second of CPU time here. It is interesting to note that *u*_*g*,*max*_ and *u*_*o*_ mainly contribute to the variance of *t*_*g*_(***X***), while *u*_*g*,*max*_, *u*_*z*,*max*_, and *u*_*o*_ are the significant contributors to the variance of *t*_*z*_(***X***).

**Fig 5:**
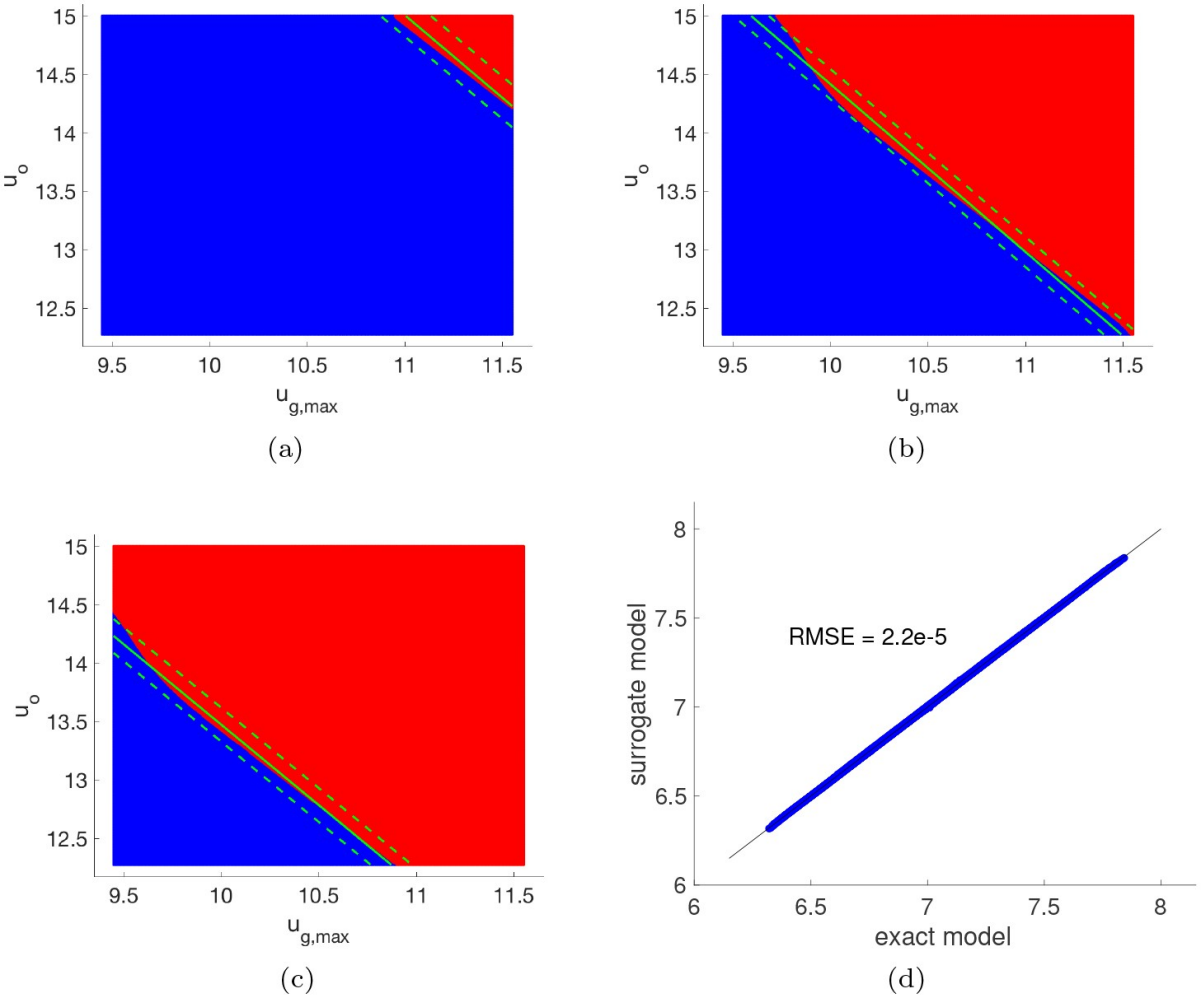
Parameter space decomposition over time. The decomposition of the parameter support into two non-overlapping elements at **(a)** 6.5 hr, **(b)** 7.0 hr, and **(c)** 7.25 hr using a sparse PCE model of the glucose singularity time *t*_*g*_. The blue and red regions represent parameters for which *t*_*g*_(***x***) > *t* and *t*_*g*_(***x***) ≤ *t*, respectively, projected onto the two most sensitive parameters. The green line represents the decision boundary learned using an SVM classifier trained with the same set of data as the sparse PCE model, while the dashed green lines represent the corresponding 95% confidence limits. **(d)** Parity plot for the sparse PCE model of *t*_*g*_ for 1e4 validation points.

The surrogate models can also be used to estimate the PDF of *t*_*g*_(***X***) and *t*_*z*_(***X***), as shown in Fig 4. From the estimated PDFs, we find that *t*_*g*_(***X***) ranges from approximately 6.31 to 7.87 hr, whereas *t*_*z*_(***X***) ranges from approximately 7.33 to 9.12 hr. This suggests that the model response is a non-smooth function of ***X*** ∈ *S* for any *t* ∈ [6.31, 9.12] hr, so that we must split *S* into two disjoint regions according to 15. Since the supports of *t*_*g*_(***X***) and *t*_*z*_(***X***) partially overlap for any *t* ∈ [7.33, 7.87] hr, additional elements should be introduced to ensure the model response is smooth. However, for times outside of this window, we can exclusively define the elements of the parameter space in terms of *t*_*g*_ for times before 7.33 hr and *t*_*z*_ for times after 7.87 hr. Plots of these two regions at times 6.5, 7.0, and 7.25 hr projected onto the two most sensitive parameters are shown in Fig 5. The blue region represents *S*_1_ while the red region represents *S*_2_. For comparison purposes, we also show the decision boundary in green (along with 95% confidence limits with dashed green lines) learned from a support vector machine (SVM) binary classifier [46] that was trained using the same 500 data points. The SVM model is unable to capture the significant nonlinear behavior of the boundary as it evolves over time. Thus, SVM results in relatively large misclassification errors due to the limited training data. The sparse PCE model, however, is able to accurately represent the *t*_*g*_ function over the full support (see the parity plot in Fig 5d), which leads to a much more accurate representation of these two elements using limited data.

The “true” RMSE values reported in Fig 3 were estimated using a large validation set that consisted of 10,000 evaluations of the full DFBA model, which required over 3 hours of CPU time. Ideally, these additional model evaluations could be avoided by directly estimating the RMSE from the ED either empirically or using cross-validation techniques. The empirical estimate of the RMSE is based on sample-based approximations to the integral expressions for mean and variance. Cross-validation obtains a more robust RMSE estimate by splitting the ED into various training and validation sets, fitting different models with each training set, and averaging the prediction error of each model. We focus exclusively on *ε*_*LOO*_ in this work. Table 1 gives the estimated RMSE values for the sparse PCE surrogate models fit using different ED sizes. We observe that the empirical estimator greatly underpredicts the “true” RMSE found from the large validation set. In fact, for the smallest size *N* = 10, the empirical estimate is a factor of 10^4^ smaller than the true RMSE. The cross-validated RMSE, on the other hand, predicts the correct order in all considered cases except *N* = 10 where it is off merely by a factor of 10 instead of 10^4^. Note that *ε*_*LOO*_ is used within the hybrid LAR algorithm to select the best surrogate out of all potential candidates.

**Table 1:** Relative mean square error estimates for glucose singularity time surrogate models under multiple experimental design sizes.

*N*	Validation	Cross-validation	Empirical
10	1.014e-02	1.268e-03	2.601e-06
50	2.230e-04	3.718e-04	2.130e-04
100	1.616e-04	1.347e-04	5.333e-05
150	7.864e-05	6.468e-05	2.820e-05
200	5.787e-05	3.898e-05	2.416e-05
500	1.817e-05	1.273e-05	7.679e-06

The validation error is computed using a large set of samples not used in the fitting procedure. Cross-validation and empirical error, however, are computed using only points in the original experimental design. Cross-validation partitions the experimental design into various training and validation sets such that multiple models can be fit and their prediction errors averaged in order to compute more robust error estimates than its empirical counterpart. Here, a leave-one-out cross-validation procedure is utilized.

#### Validation of nsPCE surrogate models

We have verified that the PCE surrogate models are able to accurately represent the singularity manifold that leads to non-smooth behavior in the states of the DFBA model. Thus, they can be used to build nsPCE surrogates for the extracellular concentrations based on the algorithm summarized in Fig 1. We choose three quantities of interest for illustrative purposes: glucose at time 7.0 hr, xylose at time 8.0 hr, and biomass at time 8.0 hr. We look to compare so-called global PCE to the proposed nsPCE method for these three quantities of interest. In global PCE, a single surrogate model is constructed over the full parameter support, while nsPCE systematically breaks down the support into two disjoint elements using the singularity time function as a dividing boundary. To ensure a fair comparison, the expansion coefficients of both global PCE and nsPCE are estimated using the basis-adaptive hybrid LAR strategy with maximum degree varying from 1 to 20 and *q* = 0.75 in the hyperbolic truncation scheme (10). In addition, the ED in both approaches are sequentially enriched using MC sampling with a fixed random seed. To simplify the construction of the polynomial basis functions when training the nsPCE surrogate models, the elements *S*_1_ and *S*_2_ were outerbounded with hyper-rectangles. However, only parameter values that explicitly fall within these sets are incorporated into the local ED. This simple approach for dealing with elements of any shape is currently used in the provided scripts [29], but other ways of dealing with generic elements can also be explored.

**Fig 6:**
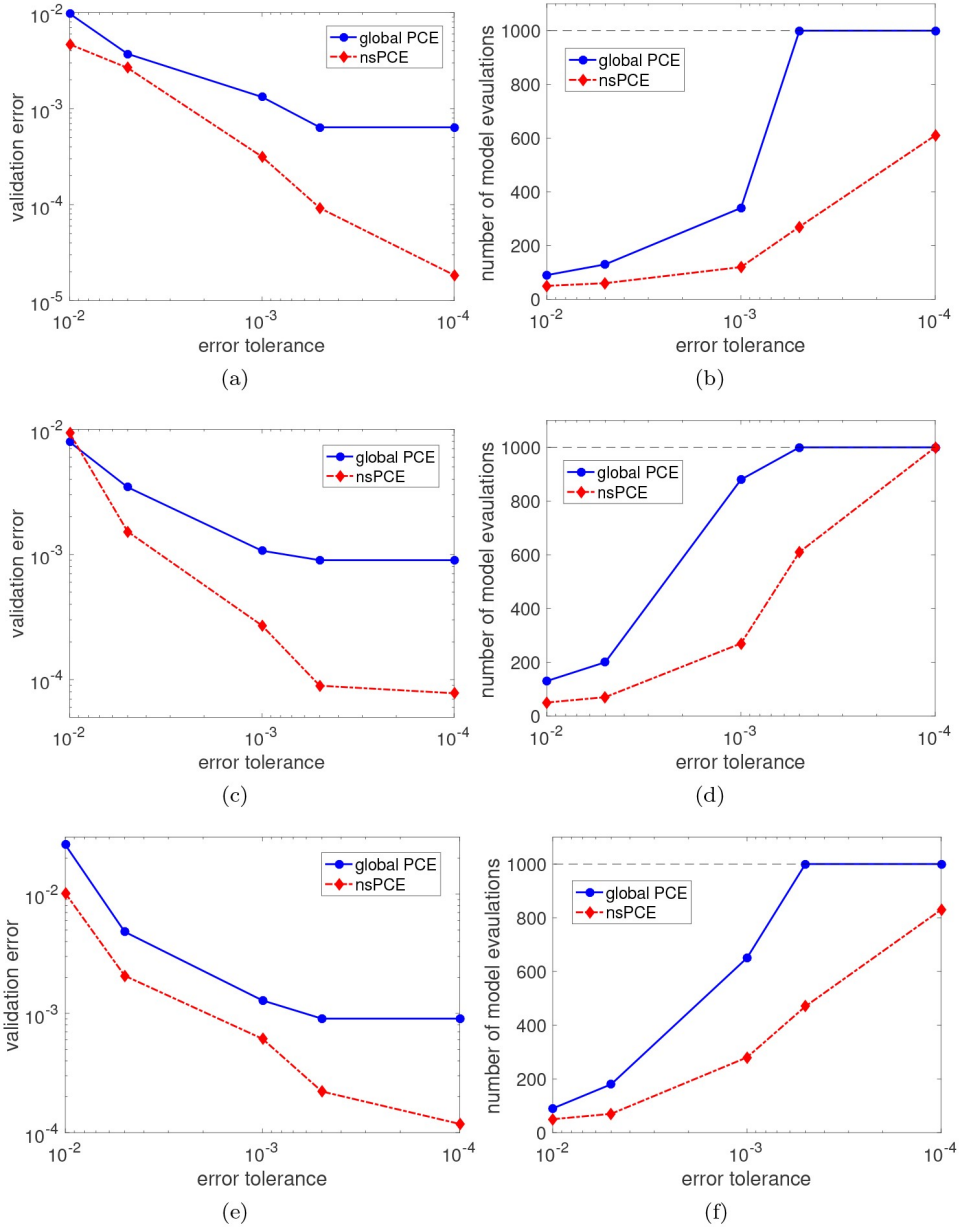
Convergence properties of nsPCE surrogate models. **(a,b)** Glucose concentration at time 7 hours. **(c,d)** Xylose concentration at time 8 hours. **(e,f)** Biomass concentration at time 8 hours. Left plots show the validation RMSE versus the specified error tolerance. Right plots show the total number of model evaluations based on a sequential ED construction, with a maximum of 1000 samples allowed. The global sparse basis-adaptive PCE results are also shown for comparison purposes.

**Fig 7:**
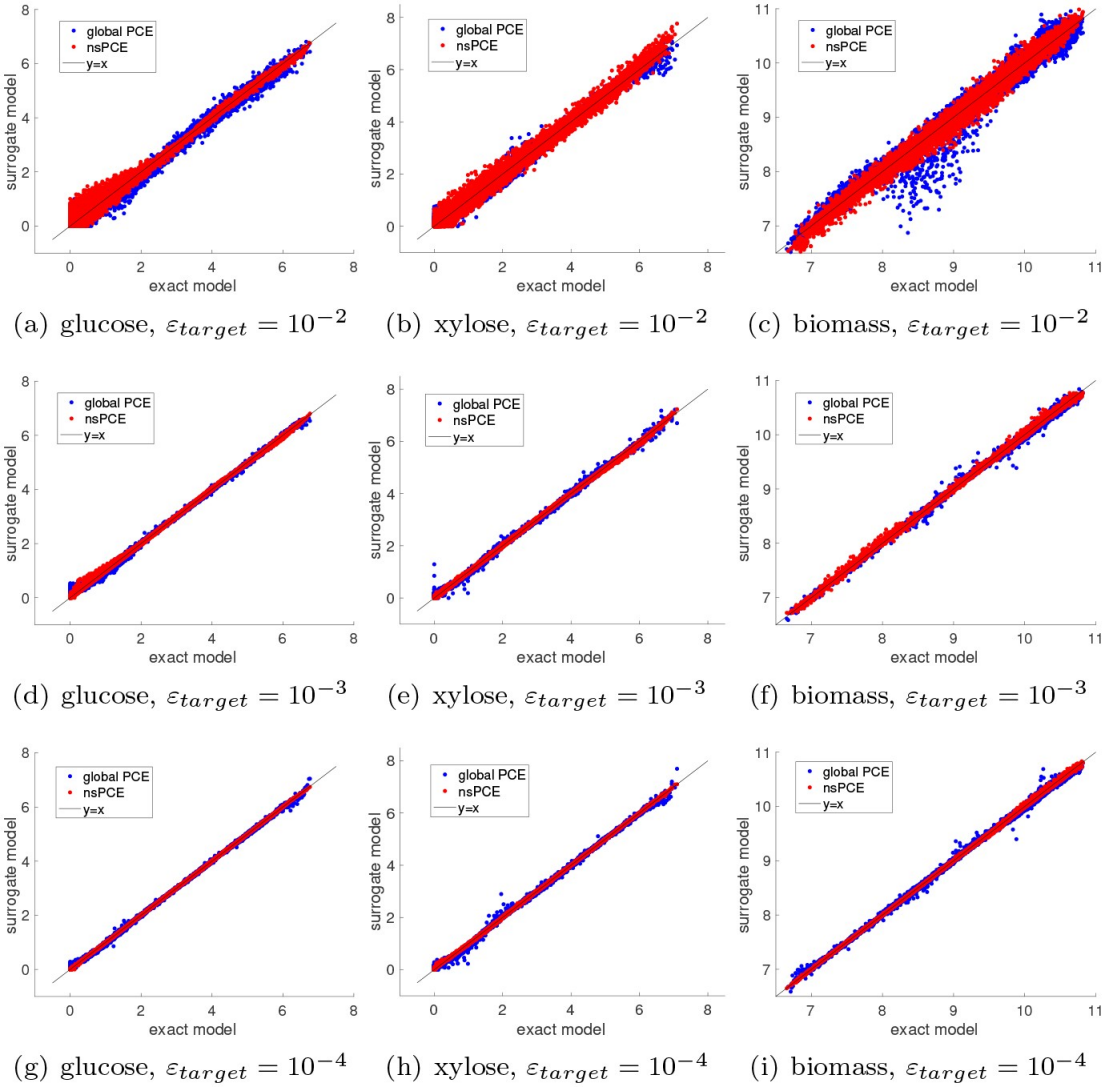
Parity plots for nsPCE surrogate models. **(a,b,c)** Target RMSE level *ε*_*target*_ = 10^−2^. **(d,e,f)** Target RMSE level *ε*_*target*_ = 10^−3^. **(g,h,i)** Target RMSE level *ε*_*target*_ = 10^−4^. The left, middle, and right columns correspond to glucose concentration at 7 hours, xylose concentration at 8 hours, and biomass concentration at 8 hours, respectively. The parity plots for global sparse basis-adaptive PCE are overlaid for comparison purposes. The global PCE has considerably larger error than nsPCE.

The convergence properties of the nsPCE surrogate models for the three quantities of interest are compared to that of global PCE in Fig 6. The nsPCE surrogates achieve significantly lower RMSE than the global PCE surrogates in virtually all cases considered, while requiring many fewer samples to converge to the target error level. In addition, global PCE saturates at the maximum number of ED samples for all three quantities of interest. This implies that global PCE is unable to achieve the desired accuracy levels, whereas nsPCE only saturates for the lowest target error of xylose. This behavior is expected since the convergence rate of global PCE is known to be substantially lowered whenever singularities are present in the model response function. Thus, nsPCE is able to significantly improve the rate of convergence based on a properly chosen elemental decomposition of the parameter support. To show that lower target error levels translate to improved predictions, parity plots for the three quantities of interest are shown in Fig 7. Note that global PCE has large prediction errors for particular values of the parameters (see the blue dots that largely deviate from the *y* = *x* line), which is likely due to the fact that an inherently non-smooth function is being represented by smooth polynomials. This is highly undesirable when using the PCE to predict *specific* response values, as opposed to predicting statistical quantities that average over the response values where individual points are not as important. The nsPCE surrogate models clearly mitigate this limitation of global PCE in a significant way since there are no outlier predictions in the set of 10,000 validation points.

#### Bayesian parameter inference

Here, we focus on the inverse UQ problem of estimating parameters from data, which can be greatly accelerated using nsPCE. The same data set used in [8] is utilized, which includes measurements of the extracellular biomass, glucose, and xylose concentrations at *t* ∈ {5.5, 6.0, 6.5, 7.0, 7.25, 8.0, 8.25, 8.5} hr. The measurements are corrupted with noise

$$\begin{array}{cc} D_{i}^{b}=b\left(t_{i};X\right)+E_{i}^{b}, & i=1,\ldots ,8,\\ D_{i}^{g}=g\left(t_{i};X\right)+E_{i}^{g}, & i=1,\ldots ,8,\\ D_{i}^{z}=z\left(t_{i};X\right)+E_{i}^{x}, & i=1,\ldots ,8, \end{array}$$ (21)

where $D_{i}=\left(D_{i}^{b},D_{i}^{g},D_{i}^{z}\right)$ and $E_{i}=\left(E_{i}^{b},E_{i}^{g},E_{i}^{z}\right)$ are, respectively, vectors of the measured data and noise at the *i*th time point. The concatenated data (respectively noise) vector is denoted by ***D*** = (***D***_1_, …, ***D***_8_) (respectively ***E*** = (***E***_1_, …, ***E***_8_)). The measurement noise variables are modeled as independent zero-mean Gaussian random variables with state-dependent variance that equals 5% of the measured signal, i.e.,

$$\begin{array}{c} E_{i}^{v}∼N\left(0,\sigma_{v,i}^{2}\left(X\right)\right),\;\;\sigma_{v,i}\left(X\right)=0.05\left|v\left(t_{i};X\right)\right|,\;\;v\in \left\{b,g,z\right\}. \end{array}$$ (22)

Given a set of measurements, the change in the state of information about the parameters is given by Bayes’ rule [11]

$$\begin{array}{c} f_{X|D}\left(x|d\right)=\frac{f_{D|X}\left(d|x\right)f_{X}\left(x\right)}{f_{D}\left(d\right)}, \end{array}$$ (23)

where *f*_***X***|***D***_ is the posterior density; *f*_***D***|***X***_ is the likelihood function; *f*_***X***_ is the prior density; and *f*_***D***_ is the evidence. As Bayesian inference looks to characterize the full posterior density, it directly provides an explicit representation of the uncertainty in the parameter estimates.

The prior and likelihood function must be specified before solving (23). We assume the same uniform priors as those used to construct the nsPCE surrogate models, though these can differ in general. The likelihood function describes the discrepancy between the observed data and the model predictions in a probabilistic way. The likelihood function is specified by the data and noise models in (21) and (22), and is given by

$$\begin{array}{c} f_{D|X}\left(d|x\right)=\prod_{i=1}^{8}\prod_{v\in \{b,g,z\}}\;\frac{1}{\sqrt{2\pi \sigma_{v,i}^{2}\left(x\right)}}\text{exp}\;(-\frac{{\left(d_{i}^{v}-v\left(t_{i};x\right)\right)}^{2}}{2\sigma_{v,i}^{2}\left(x\right)}). \end{array}$$ (24)

Although we use a Gaussian likelihood here, the same Bayesian estimation approach can be applied to any choice of likelihood function and thus can be easily modified to incorporate other potentially important factors including sensor bias or asymmetric noise.

Since (23) cannot be solved analytically, we must resort to sample-based approximations that rely on generating samples from the target posterior distribution. A variety of methods have been developed for sampling from the unknown posterior *f*_***X***|***D***_, including Markov Chain Monte Carlo (MCMC) [47–49] and sequential Monte Carlo (SMC) [50–52] algorithms. The proposed surrogate models can be used to accelerate any sampling-based method; however, we focus on SMC since this is a class of algorithms that can be made fully parallelized. SMC is based on the concept of *importance sampling*, which can be implemented in an iterative fashion such that the posterior is updated every time a new measurement becomes available. For a given number of particles *N*_*p*_, the SMC approximation to (23) can be summarized as follows:

1. Initialization: set *k* = 1 and generate samples and weights ${\left\{x_{i},w_{i}\right\}}_{i=1}^{N_{p}}$ from prior.
2. Reweighting: update the weights *w*_*i*_ ← *w*_*i*_ × *w*_*k*_(***x***_*i*_) where *w*_*k*_(***x***_*i*_) ∝ *f*_*D*_*k*_|***X***_(*d*_*k*_|***x***_*i*_).
3. Resampling: resample ${\left\{x_{i},w_{i}\right\}}_{i=1}^{N_{p}}$ for particles with equal weights ${\left\{x_{i}^{r},\frac{1}{N_{p}}\right\}}_{i=1}^{N_{p}}$.
4. Loop: set *k* ← *k* + 1 and ${\left\{x_{i},w_{i}\right\}}_{i=1}^{N_{p}}\leftarrow {\left\{x_{i}^{r},\frac{1}{N_{p}}\right\}}_{i=1}^{N_{p}}$. Return to Step 2 if *k* < *k*_*f*_.

When the algorithm stops at time *k*_*f*_, the set of *N*_*p*_ particles targets the posterior distribution of interest. We use systematic resampling in Step 3 due to its computational simplicity and good empirical performance, though a variety of other methods are available [50]. Step 2 is usually the computational bottleneck because the model must be repeatedly solved in order to evaluate the likelihood weight factors using (24). Therefore, we propose to replace the evaluation of *v*(*t*_*i*_; ***x***) with a nsPCE surrogate model *v*^*nsPCE*^(*t*_*i*_; ***x***) for every *v* ∈ {*b*, *g*, *z*} and *i* = 1, …, 8. We must then construct a total of 24 surrogates before running the SMC algorithm.

The same basic strategy described in the previous section is used for constructing all 24 of the nsPCE surrogate models. Similarly to how the samples for the singularity time are used to initialize the ED in each element, we can store the list of state and time points generated when integrating the DFBA model and interpolate these points to calculate the extracellular concentrations at every time point of interest. By keeping a working ED that is used to initialize each element at every time point, we can greatly limit the number of expensive DFBA simulations that represent the computational bottleneck in SMC. The proposed algorithm in Fig 1 is run with a target error of *ε*_*target*_ = 10^−3^, 250 initial ED samples, 10 ED samples added at each iteration, 2500 maximum ED samples, maximum degree varying from 1 to 20, and hyperbolic truncation with *q* = 0.75. The algorithm converged with cross-validated errors *ε*_*LOO*_ below the desired tolerance using only a total of 1200 DFBA simulations to train all 24 nsPCE surrogate models. The basis-adaptive hybrid LAR method consistently estimated coefficients in less than 30 seconds, verifying that the DFBA simulations are the dominant cost in this case study. The validation RMSE values are summarized in S2 Table, which are all below the target error threshold.

**Fig 8:**
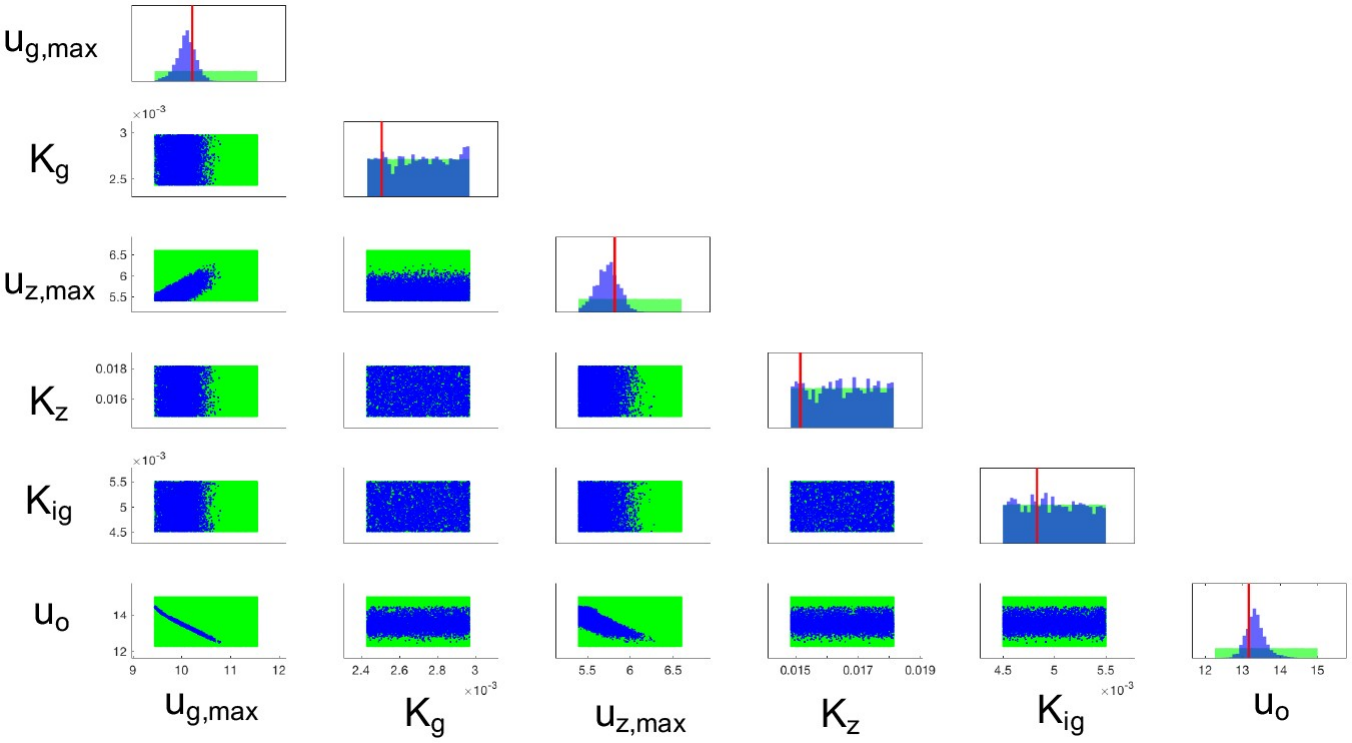
Posterior distribution of the estimated model parameters. The diagonal subplots represent marginal densities while the off-diagonal subplots represent two-dimensional projections of samples from the joint density. Blue denotes the posterior density while green denotes the prior density. The red line represents the true parameter values used to generate synthetic data for estimation purposes.

Fig 8 shows the posterior density estimated using SMC with *N*_*p*_ = 1 × 10^6^ particles for a synthetic data set, where the likelihood weights are evaluated using the inexpensive nsPCE surrogate models. The synthetic data (‘x’ marks in S1 Fig) was obtained by simulating the genome-scale *E. coli* DFBA model with fixed parameters (red lines in Fig 8) and adding random noise realizations (22) to the resulting model outputs. The 1200 DFBA simulations used to construct the surrogates require ∼ 30 minutes of CPU time while the surrogate-based SMC algorithm, which takes advantage of vectorization, finishes in ∼ 2 minutes of CPU time. Hence, over 800-fold savings in computational cost is achieved when compared to SMC without surrogates that would require approximately 17 days of CPU time under the same settings (1 × 10^6^ DFBA simulations at a cost of 1.5 seconds per evaluation). The DFBA model predictions under the MAP estimates (i.e., parameters that maximize the posterior) are shown in S1 Fig, which closely match the observed data. To verify that the SMC algorithm approximately converged with this many particles, we performed 10 separate bootstrap runs that produced a set of very similar posterior densities. Note that a discussion on challenges and open issues in Bayesian estimation is provided in the Discussion section. The SMC code is provided in the main_smc.m script in [29].

**Fig 9:**
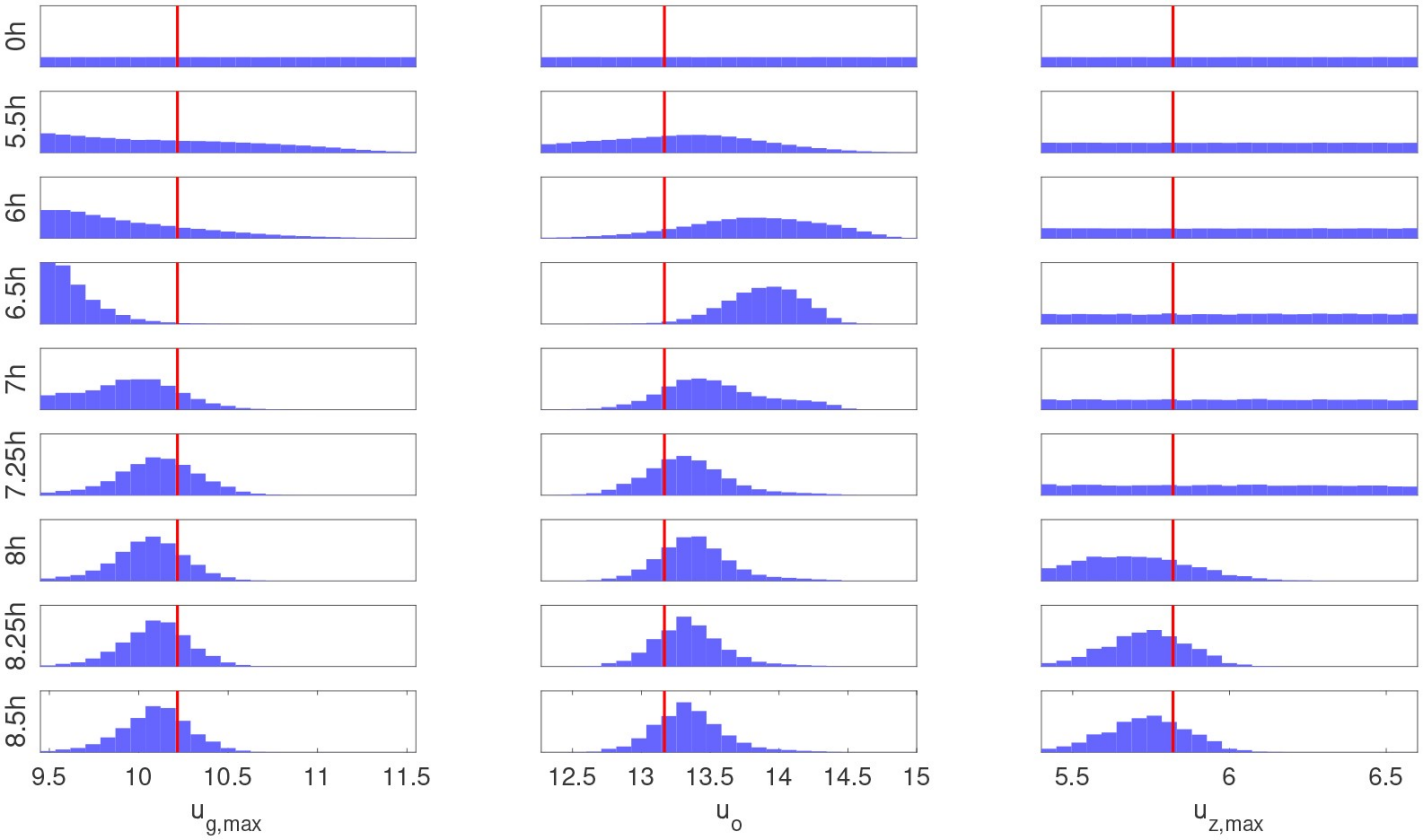
Evolution of the posterior marginal densities for the observable model parameters over time. Each subplot shows the histogram of parameter posterior samples estimated using the sequential Monte Carlo method. The *x*-axis represents the range of values of the parameters and the *y*-axis represents frequencies. The red line represents the true parameter values.

The estimated posterior density in Fig 8 provides interesting physical insights. Three of the parameters (*K*_*g*_, *K*_*z*_, *K*_*ig*_) are unobservable with the current data set since their posterior (blue) and prior (green) densities are equivalent. This observation could not be easily made before running the estimation procedure due to the nonlinear and indirect relationship between ***D*** and ***X***. A change in the experimental conditions such as the initial conditions, controlled oxygen concentration, or substrate feed profiles can enhance the sensitivity of the data to parameters (*K*_*g*_, *K*_*z*_, *K*_*ig*_). For example, running the batch at low glucose concentrations *g*(*t*) ≪ *K*_*g*_ results in a glucose uptake rate $u_{g}\left(t\right)\approx u_{g,max}\frac{g(t)}{K_{g}}$ that is a strong function of *K*_*g*_, whereas running the batch at high glucose concentrations (as done in this case study) produces a nearly constant uptake rate *u*_*g*_(*t*) ≈ *u*_*g*,*max*_ that is independent of *K*_*g*_. Although the data is sensitive to (*u*_*g*,*max*_, *u*_*z*,*max*_, *u*_*o*_), these parameters are highly correlated as seen in the off-diagonal plots of their joint densities in Fig 8. Thus, the currently available data from one single batch is insufficient for accurately estimating all the parameters of interest. The evolution of the marginal posterior densities of the observable parameters over time is shown in Fig 9. Since glucose is mostly consumed by 7.25 hr, the densities of *u*_*g*,*max*_ and *u*_*o*_ remain constant for the remaining batch time. The density of *u*_*z*,*max*_, however, is constant before 7.25 hr because xylose remains mostly at its initial condition.

#### Forward uncertainty propagation

**Fig 10:**
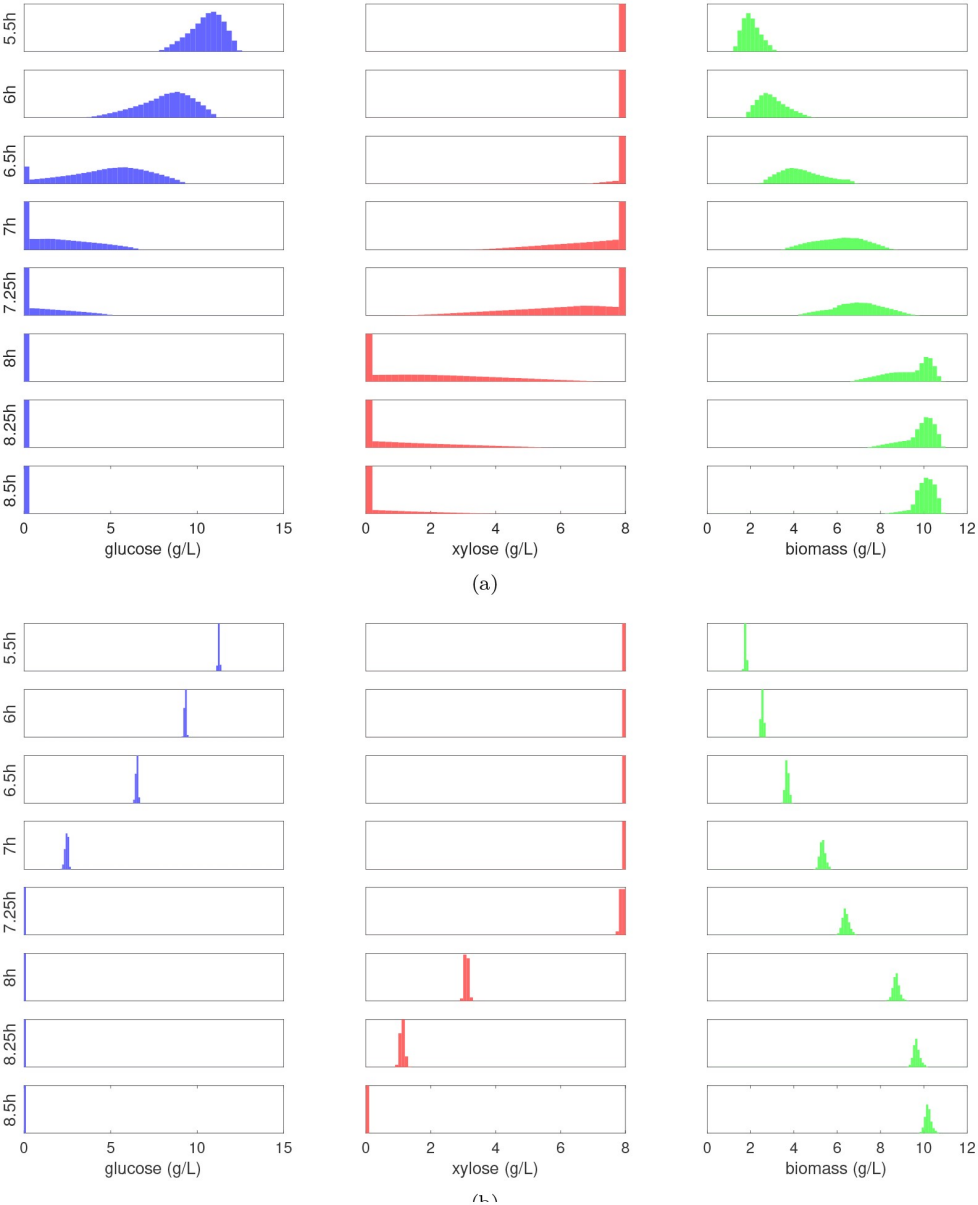
Predicted probability distributions of extracellular concentrations. **(a)** Model predictions using parameter samples from the prior. **(b)** Model predictions using parameter samples from the posterior. Each subplot shows the histogram of samples of the model output obtained by substituting i.i.d. samples from the parameter distribution into the corresponding ME-PCE surrogate model. The *x*-axis represents the range of values of the model outputs and the *y*-axis represents frequencies.

Let Y=M(X) denote the vector of all model responses. The forward UQ problem looks to characterize the uncertainty in the model predictions by propagating uncertainty in the parameters through M. This can involve estimating either the prior predictive distribution *f*_***Y***_ (before any data has been collected), or the posterior predictive distribution *f*_***Y***|***D***_ (after data has been obtained). The only difference between these two problems is that M is evaluated at i.i.d. samples drawn from the prior in the former and the posterior in the latter. The densities of the model predictions estimated using 1 × 10^6^ samples are shown in Fig 10. By replacing the full DFBA model with the nsPCE surrogate model, these histograms were obtained in less than 1 minute of CPU time. As expected, the prior predictive distributions are much wider than the posterior predictive distributions, indicating there is significant uncertainty in the predictions before incorporating data. In addition, we see that many of these distributions have sharp changes and long tails due to the non-smooth behavior of the model responses, which can be accurately captured with the proposed nsPCE framework. It is also interesting to note that the posterior predictive distributions have low variance, even though the parameters are not perfectly estimated. This highlights the impact that nonlinearity can have on both estimation and uncertainty propagation.

### Case study 2: Synthetic metabolic network

This case study is based on a synthetic metabolic network originally introduced in [31, Chapter 8]. The goal of this case study is to show that the proposed nsPCE method can be applied to problems with a larger number of parameters as well as alternative UQ approaches. The synthetic metabolic network consumes a carbon source *C*, a nitrogen source *N*, and an oxygen source *O* to produce lipids *L*, ethanol *E*, biomass *X*, *ATP*, and some oxidation product *COX*. Although used for illustrative purposes, this network is meant to mimic the behavior of living organisms in the sense that: (i) *E* can only be produced in the absence of *O*, (ii) *L* can only be accumulated in the absence of *N*, (iii) there is a minimum *ATP* requirement, and (iv) the aerobic oxidation of *C* produces more energy than fermentation of *C*. The set of reactions can be summarized as

$$\begin{array}{c} \begin{array}{cc} v_{C} & :C_{ex}\to C,\\ v_{N} & :N_{ex}\to N,\\ v_{O} & :O_{ex}\to O,\\ v_{OX} & :C+O\to S_{ATP,OX}ATP+S_{OX,OX}COX_{ex},\\ v_{Ferm} & :4C\to S_{ATP,Ferm}ATP+E_{ex}+S_{OX,Ferm}COX_{ex},\\ v_{L} & :4C+S_{ATP,L}ATP\to L,\\ v_{X} & :S_{C,X}C+S_{N,X}N+S_{ATP,X}ATP\to X,\\ v_{ATP} & :ATP\to ATP_{maintenance}, \end{array} \end{array}$$ (25)

where the subscript *ex* denotes extracellular metabolites and all of the reactions are assumed to be unidirectional. The unknown stoichiometric coefficients are denoted by *S*_*i*,*j*_, where *i* represents the metabolite name and *j* represents the reaction name. The dynamic mass balance equations for the extracellular environment are given by

$$\begin{array}{c} \begin{array}{ccc} \dot{X}\left(t\right) & =v_{X}\left(t\right)X\left(t\right), & X\left(0\right)=X_{0},\\ \dot{C}\left(t\right) & =-v_{C}\left(t\right)X\left(t\right), & C\left(0\right)=C_{0},\\ \dot{N}\left(t\right) & =-v_{N}\left(t\right)X\left(t\right), & N\left(0\right)=N_{0},\\ \dot{O}\left(t\right) & =-v_{O}\left(t\right)X\left(t\right), & O\left(0\right)=O_{0},\\ \dot{L}\left(t\right) & =v_{L}\left(t\right)X\left(t\right), & L(0)=0,\\ \dot{E}\left(t\right) & =v_{Ferm}\left(t\right)X\left(t\right), & E(0)=0,\\ \dot{COX}\left(t\right) & =(S_{OX,OX}v_{OX}\left(t\right)+S_{OX,Ferm}v_{Ferm}\left(t\right))X\left(t\right), & COX(0)=0,\\ \dot{\alpha }\left(t\right) & =\gamma (t), & \alpha (0)=0, \end{array} \end{array}$$ (26)

where *α* is a penalty state that remains equal to zero until the state trajectories become infeasible (e.g., when all of the metabolites are depleted). A detailed discussion on how to determine the instantaneous penalty value *γ* is provided in [10], which is automatically computed in DFBAlab. We assume that the uptake kinetics are given by the following expressions

$$\begin{array}{c} \begin{array}{cc} v_{C}^{UB}\left(s\right) & =\text{max}\;(0,v_{max,C}\frac{C}{K_{C}+C}\frac{1}{1+\frac{E}{K_{iE}}}),\\ v_{N}^{UB}\left(s\right) & =\text{max}\;(0,v_{max,N}\frac{N}{K_{N}+N}),\\ v_{O}^{UB}\left(s\right) & =\text{max}\;(0,v_{max,O}\frac{O}{K_{O}+O}), \end{array} \end{array}$$ (27)

where **s** = (*X*, *C*, *N*, *O*, *L*, *E*, *COX*, *α*) is the vector of extracellular species. A hierarchical set of objectives is used in the FBA problem (2) to ensure that unique reaction fluxes are obtained (see S3 Table). A total of twenty parameters in this DFBA model, appearing in both intracellular and extracellular quantities, are assumed to be uniformly distributed between upper and lower bounds summarized in S4 Table.

#### Global sensitivity analysis

**Fig 11:**
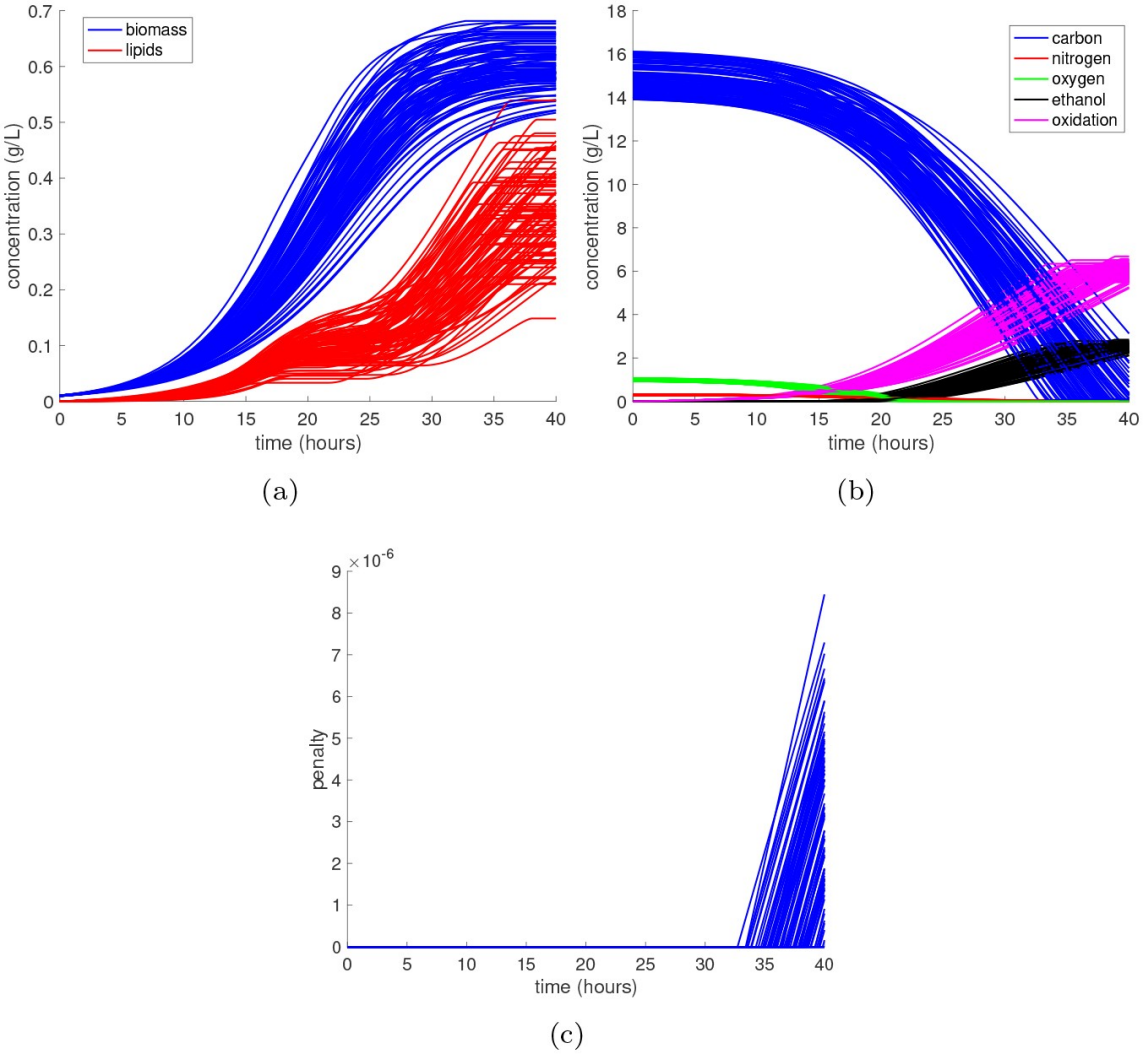
Monte Carlo simulation of the synthetic metabolic network. The synthetic DFBA model with twenty uncertain parameters is integrated from time 0 to 40 hours for 100 different parameter realizations drawn independently from the uniform prior density. The time profiles are shown for **(a)** biomass and lipids, **(b)** the substrates and products, and **(c)** the penalty state.

To locate any possible singularities, we first simulate the DFBA model with randomly sampled parameter values. The results are shown in Fig 11 wherein we see that the penalty state becomes positive *α*(*t*) > 0 once all substrates are depleted, which introduces a strong discontinuity into the state profiles. Even though this is a considerably smaller metabolic network than the one considered in the *E. coli* case study, it still takes approximately 0.5 seconds of CPU time per realization of the parameter. Thus, it is still advantageous to construct a surrogate model to speedup both forward and inverse UQ problems.

We look to run the proposed nsPCE method (see Fig 1) using the time that the penalty state switches from zero to positive as the singularity time. As suggested in [31, Chapter 8], we consider the seven substrate and product concentrations (*X*, *C*, *N*, *O*, *L*, *E*, *COX*) at four time points *t* ∈ {10, 20, 30, 40} hr as our main quantities of interest. The nsPCE method was applied in the same manner as described in the previous case study. Here, we specified a target error of *ε*_*target*_ = 10^−3^, 100 initial ED samples, 100 ED samples added at each iteration, 1500 maximum ED samples, the maximum polynomial degree could vary from 1 to 30, and a hyperbolic truncation scheme with *q* = 0.6. The algorithm converged using a total of 2800 DFBA simulations. The resulting parity plots are shown in S2 Fig, which all have empirical RMSE values significantly below the target error. To further assess the accuracy of these models, we calculated the RMSE using 1000 additional samples that were not used during the training process. The validation RMSE averaged over the 28 models was found to be 8.1 × 10^−4^. Only 6 of the 28 surrogates had RMSE values slightly above the target, with the largest overall RMSE being 3.5 × 10^−3^, indicating that the surrogates are reasonably accurate representations of the original model. Note that these errors can be refined by specifying a lower *ε*_*target*_ at the cost of more DFBA simulations.

**Fig 12:**
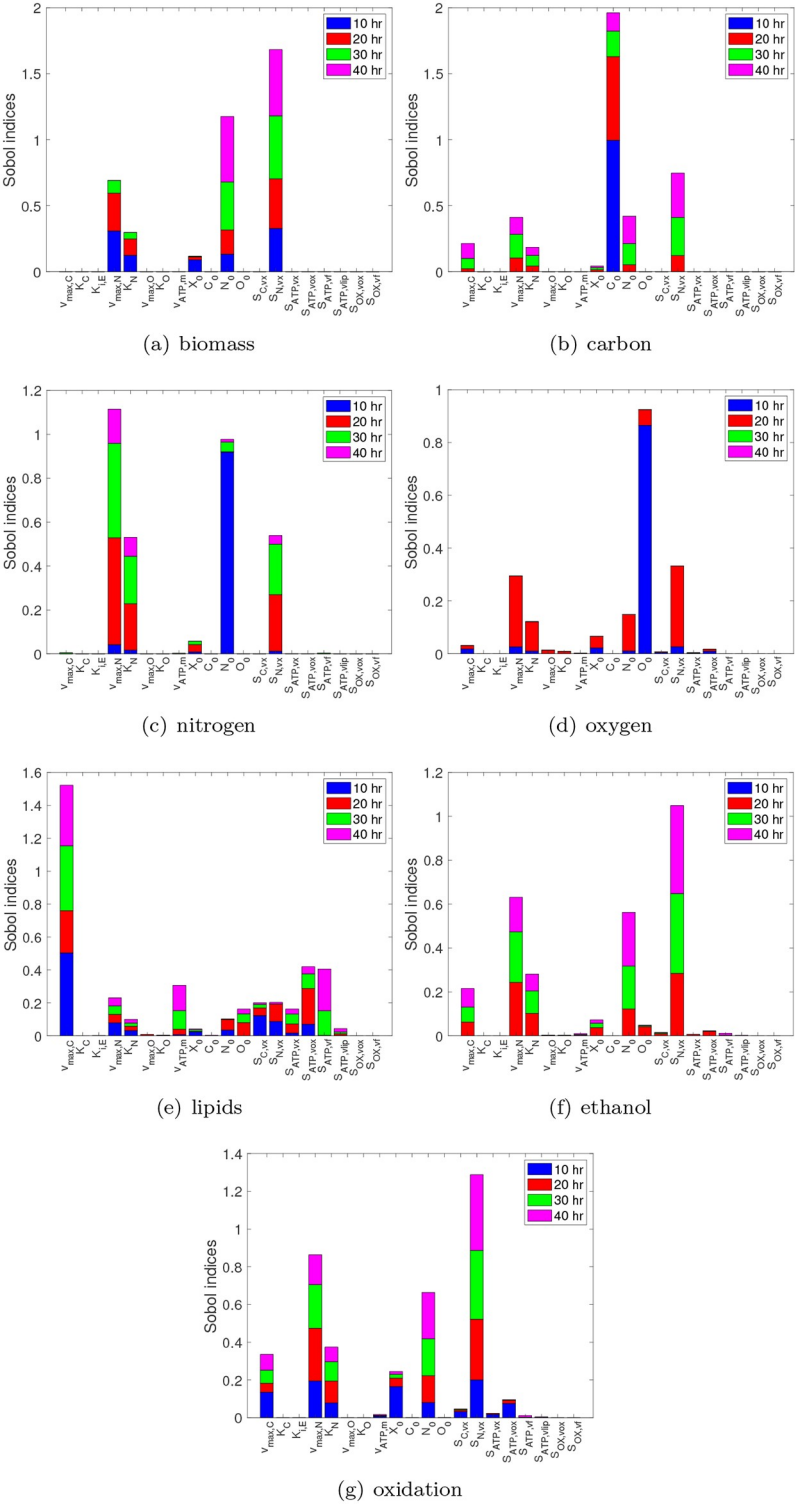
Global sensitivity indices for the quantities of interest in the synthetic metabolic network. **(a)**-**(g)** Global sensitivity indices for extracellular substrate and product concentrations at various time points with respect to the twenty uncertain parameters.

Once the nsPCE surrogate models are constructed, they can be used to efficiently perform global sensitivity analysis in order to quantify the respective effects of each individual parameter on the variance of the model response. Although many sensitivity measures exist, we use Sobol’ indices since they make no assumption on the underlying linearity or monotonicity of the model. The global sensitivity results for the various quantities of interest over time are shown in Fig 12. A variety of interesting conclusions can be drawn from these results. For example, the model appears to be insensitive to *K*_*C*_ and *K*_*iE*_, which is likely due to the fact that the batch was run at high carbon and low ethanol concentrations. In addition, the measurements of carbon, nitrogen, and oxygen are highly sensitive to their respective initial conditions *C*_0_, *N*_0_, and *O*_0_ at the first measurement time of 10 hr; however, this sensitivity drops considerably as time evolves. We emphasize that obtaining such insights using random sampling on the full model can be prohibitively expensive, but requires negligible cost using the nsPCE surrogate models.

#### Optimization-based parameter estimation

We now utilize maximum a posteriori (MAP) estimation to estimate the unknown model parameters from synthetically-generated experimental data (see S5 Table). The MAP estimate is defined as the mode of the posterior distribution, and can be stated directly as an optimization problem of the form [53]

$$\begin{array}{c} {\hat{x}}_{\text{MAP}}\left(d\right)=\underset{x\in S}{\text{argmax}}\;f_{X|D}\left(x|d\right)=\underset{x\in S}{\text{argmax}}\;f_{D|X}\left(d|x\right)f_{X}\left(x\right), \end{array}$$ (28)

where the prior acts as a regularization term that can stabilize the solution whenever the parameters cannot be uniquely inferred from the available data [54]. We consider a Gaussian likelihood, with noise standard deviations reported in S5 Table, and a Gaussian prior whose mean is equal to the midpoint of the bounds in S4 Table and standard deviations equal to 10% of the mean values. Under the Gaussian restrictions, we can convert the MAP problem to the minimization of a regularized weighted least squares objective by applying a negative log transformation. We solved the optimization (28) using both the full DFBA and nsPCE surrogate models in order to assess the computational gains afforded by the nsPCE method. To ensure a fair comparison, we solved both of these MAP problems in Matlab using the non-smooth optimizer SolvOpt [55] with default parameter settings and the mean of the prior as the initial guess. The algorithm took approximately 2.5 hr to converge when using the full DFBA model, which was substantially reduced to less than 2 minutes (i.e., a factor of 60) when the full model was replaced with the nsPCE surrogates.

**Fig 13:**
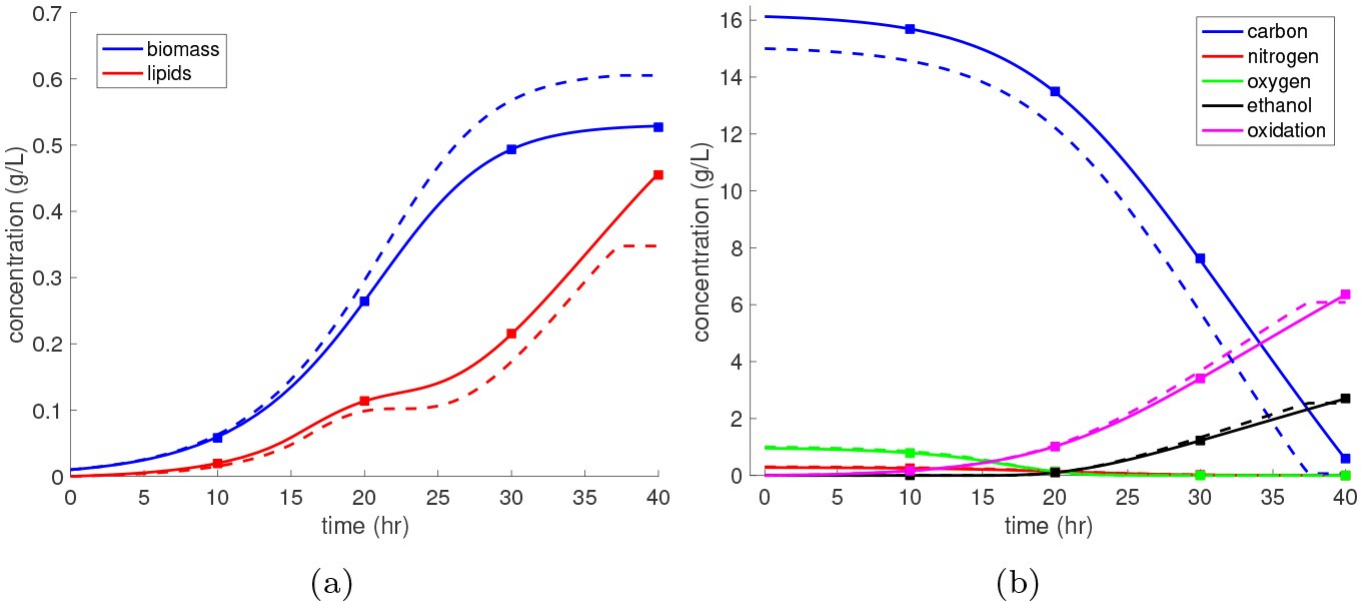
Comparison of model predictions and data for the synthetic metabolic network. The model predictions for **(a)** biomass and lipids and **(b)** the substrates and products, shown with solid lines, were obtained by integrating the DFBA model with the MAP estimates of the parameters. The ‘□’ marks represent the data that was obtained by corrupting the model predictions using the true (unknown) parameters with randomly generated noise. The dotted lines represent the model predictions based on the initial parameter guess that was used to initialization the optimizer.

Not only did the use of the nsPCE surrogate models accelerate the optimization, it also produced a solution with a lower overall objective function value. The objective improved from 471.73 to 1.56 when using the surrogate models as compared to 63.57 when using the full DFBA model. Convergence to a suboptimal solution is likely a consequence of numerical issues related to the stability of derivative approximation using finite difference in DFBA models, which were also observed in [31, Chapter 8]. On the other hand, since the nsPCE surrogate models are defined in terms of simple polynomial functions, the finite difference derivative approximation seems to produce more stable iterations towards the solution of the MAP problem, at least in this particular case study. The predictions of the DFBA model under the MAP estimates found using the nsPCE surrogates are shown in Fig 13. We see that the predictions using the posterior parameter estimates very closely match the observed data, which is a large improvement when compared to the predictions based on the prior parameter estimates.

#### Posterior distribution analysis

**Fig 14:**
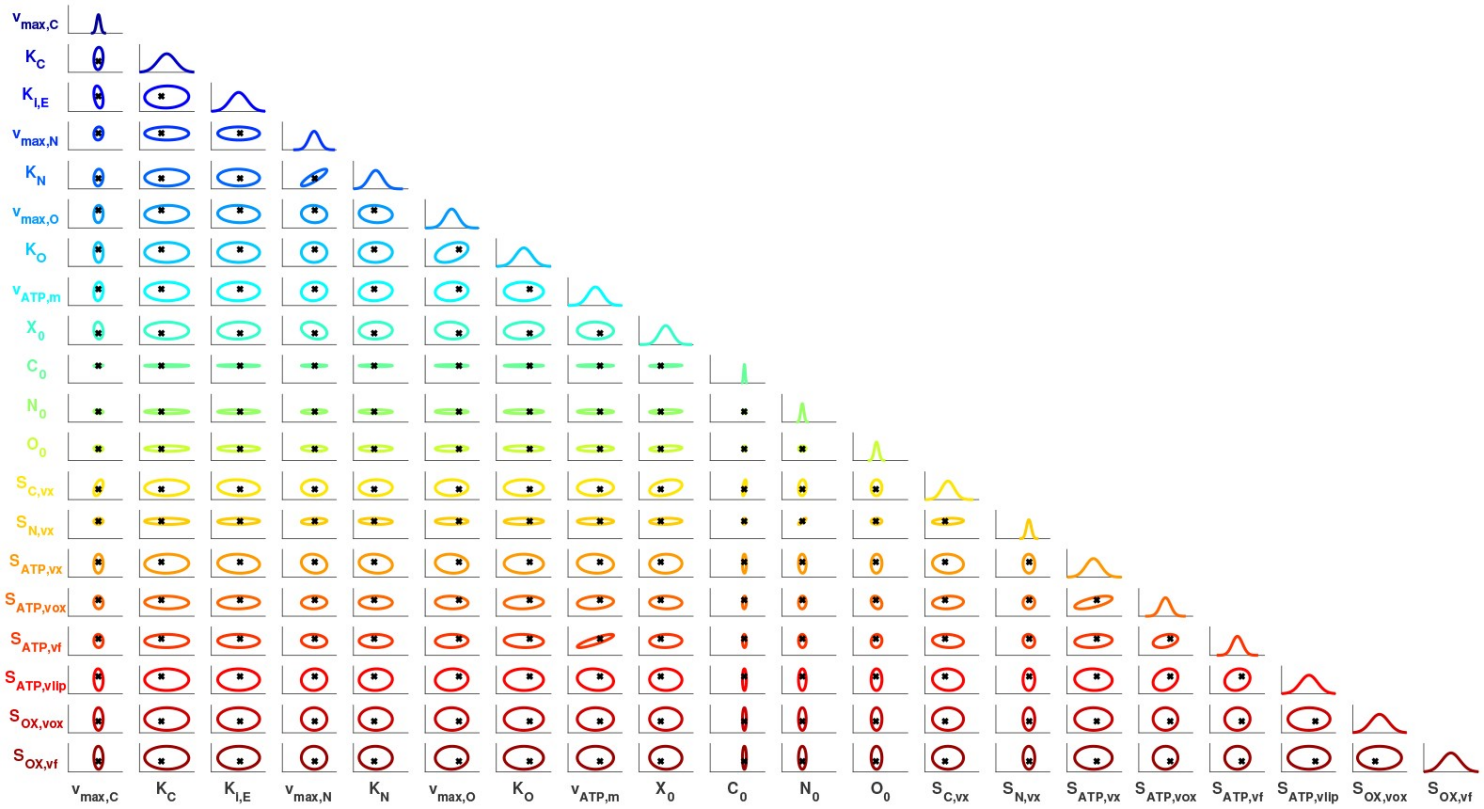
Estimated posterior distribution for the parameters of the synthetic metabolic network. The diagonal subplots represent the estimated marginal densities, while the off-diagonal subplots represent the two-dimensional projections of the 95% confidence regions. Black ‘x’ marks represent the true parameter values, while the modes of the marginal densities signify the MAP estimates.

The MAP estimation (28) determines the parameters that maximize the posterior density. However, we need a characterization of the entire posterior to assess uncertainty in these estimates. Here, we use a Laplace approximation of the posterior density, which is based on a second-order Taylor series of −log(*f*_***X***|***D***_(***x***|***d***)) around the MAP estimate [56]. As shown in [57], this leads to a Gaussian approximation of the posterior whose mean is equal to the MAP estimate and whose covariance is defined in terms of the model response sensitives. The approximated posterior marginal densities and 95% confidence regions for the twenty MAP parameter estimates are shown in Fig 14. As can be seen, the true (unknown) parameter values are contained within the reported confidence regions. We also see that the parameters with the highest global sensitivity indices (see Fig 12) are accurately estimated, whereas the parameters that have little-to-no sensitivity to the data have much wider variances that are similar to that of the prior. Lastly, we observe that physically-related parameters exhibit a significant degree of correlation including, for example, nitrogen uptake parameters *v*_*max*,*N*_ and *K*_*N*_. It is worth noting that the surrogate models can also enable the use of more advanced methods for posterior characterization such as randomized MAP [58], which would require the repeated solution of (28) with randomly perturbed data.

## Discussion

In this work, we develop a novel surrogate modeling method for handling the non-smooth nature of computationally expensive dynamic flux balance analysis (DFBA) models. It is shown that surrogate models can vastly accelerate uncertainty quantification (UQ) tasks, such as calibrating the model with experimental data (inverse problem) and quantifying confidence in the model predictions (forward problem). The proposed surrogate modeling method is based on an extension of polynomial chaos expansion (PCE), which we refer to as non-smooth PCE (nsPCE). The main idea behind nsPCE is to systematically partition the parameter space into two non-overlapping regions (or elements) on which the model response behaves smoothly. The nsPCE uses a model of the time that the singularity occurs in order to define the boundary between these two elements. State-of-the-art (i.e., sparse basis-adaptive) regression methods are used to estimate the coefficients of the expansions, such that the overall model response is approximated by a sparse piecewise polynomial function.

We demonstrate the advantages of the nsPCE surrogate modeling method on two separate case studies. The first case study is based on a DFBA model of an *E. coli* fermentation reactor under aerobic growth in a glucose and xylose mixed media. A genome-scale metabolic network reconstruction with 1075 reactions and 761 metabolites is used to represent the intracellular behavior, which results in an expensive-to-simulate DFBA model that is prohibitive for use in most UQ tasks. Thus, we illustrate how both inverse and forward UQ can be significantly accelerated using nsPCE surrogate models on this problem. In particular, we use a Bayesian estimation method to infer six uncertain parameters related to the substrate uptake kinetics from data. The posterior parameter distribution is estimated using sequential Monte Carlo with 1 × 10^6^ samples, which would have required ∼17 days of CPU time to compute using the full DFBA model, but takes less than one hour when using the nsPCE surrogate models including the cost of training the models. The resulting posterior distribution yields significant physical insights including that the available data set is insufficient to reliably estimate all six parameters, with three of the parameters being non-identifiable under the current experimental conditions. We then demonstrate the scalability of the proposed nsPCE method on a synthetic metabolic network problem with twenty unknown parameters that are related to both intracellular and extracellular quantities. We estimate these parameters using maximum a posteriori (MAP) estimation, and observe that the cost of the optimization algorithm can be reduced by a factor of 60 when using the nsPCE surrogates in place of the full DFBA model. Note that the observed speedups are expected to be even greater for more complex DFBA models, such as those with nonlinear cellular objectives, multiple cultures, or even larger metabolic networks due to the increased cost of the simulations.

### Scalability properties of nsPCE

The nsPCE method is specifically constructed to take advantage of the hybrid LAR method for sparse regression, which was originally developed in [19]. As such, nsPCE directly inherits the beneficial scalability properties of hybrid LAR that introduces two sources of sparsity into the expansions: (i) low-rank truncation that discards basis terms that lead to high-order interaction of the parameters that are irrelevant in most engineering problems and (ii) regularized least squares is used to systematically add basis terms that are strongly correlated to the model response. Additionally, the risk of over-fitting the surrogate model to the available data set can be reduced even further by making the approach basis-adaptive, i.e., separate PCE models are fit for varying maximum degrees and the one with the lowest error is selected.

The basis-adaptive hybrid LAR approach has been successfully applied to a wide-variety of problems and has consistently shown the ability to greatly mitigate the curse-of-dimensionality that is inherent in traditional PCE methods (see, for example, [19, 59, 60]). To the best of our knowledge, [60] tackled the largest problem to-date, which is a hydrogeological model with 78 parameters (68 identified to be sensitive) that can be accurately represented using a sparse PCE trained using only 2000 model evaluations. Although uncertainty in high-dimensional DFBA models has not been explored in the literature, these promising results and those shown in the synthetic case study give some confidence that nsPCE may be able to scale to the sizes needed to solve these challenging problems. Note that very recent work has shown that sparse PCEs can be applied to ultrahigh-dimensional problems (on the order of 10^4^ parameters) by incorporating a dimensionality reduction step before training the surrogate model [61]. It may be possible to use similar approaches to incorporate uncertainty in the complete set of intracellular model parameters into the nsPCE surrogate models. These are interesting and important challenges that deserve further investigation.

### Further reducing the number of model evaluations

In this work, the surrogate models are trained using experimental designs (EDs) populated with random samples of the parameters. Recent work has demonstrated that the number of ED points needed to achieve a desired accuracy level can be further reduced by maximizing the information content of the sample locations. Multiple approaches have been developed to tackle this challenging problem, including coherence-optimal sampling [62] and numerical “moment-matching” optimization [34, 37]. The optimal placement of samples in arbitrary domain shapes in a sequential fashion remains largely unexplored in the literature.

Additionally, the current implementation of nsPCE involves only two elements; however, it is unclear if the convergence rate can be improved even more by further decomposing these elements. An adaptive approach for decomposing the random parameter space that uses sensitivity information to decide which elements to split was proposed in [25]. A similar concept could be potentially utilized within nsPCE, though the method would likely benefit from the incorporation of more advanced geometries than simple boxes.

### Considerations and challenges in parameter estimation

Many of the difficulties encountered during parameter estimation are related to poor identifiability of model parameters. Performing parameter identifiability tests can help mitigate these difficulties by ensuring the parameter estimation problem is well-posed, which is especially important when dealing with limited experimental data and/or considering a large number of model parameters. It is common to distinguish between structural and practical identifiability. Structural identifiability is a theoretical property of the model structure that depends only on the observation function and the manipulated input function. Since a structurally non-identifiable parameter is independent of the accuracy of available experimental data, it cannot be resolved by a refinement of existing measurements. The only remedy is a qualitatively new measurement or experiment that alters the structure of the mapping between the parameters and the data. In contrast, practical identifiability also takes into account the amount and quality of the measured data, meaning that it can in principle be resolved by improving the quality of the measurements or increasing the number of measured time points. A thorough treatment of these issues in the context of biological models can be found in, e.g., [63–65]. To the best of our knowledge, structural and practical identifiability analysis has not been demonstrated on DFBA models, which is an interesting area for future work. It is important to note that, although many methods exist for detecting non-identifiable parameters, they often have restrictions on the class of functions so that they are not directly applicable to DFBA models.

Although not observed here, sequential Monte Carlo (SMC) can suffer from *degeneracy* wherein fewer and fewer particles retain significant weight. This is especially prevalent in high-dimensional problems including those with a large number of parameters or a large time horizon [66]. In [67], it is shown that the degeneracy phenomenon occurs unless the sample size is chosen to be exponential in the dimension, which indicates some type of curse-of-dimensionality. This sample degeneracy can be protected against by adding a *rejuvenation* step that “moves” the resampled particles according to a Markov chain Monte Carlo (MCMC) transition kernel [51]. This operation does not change the target distribution, but does reduce impoverishment since identical replicates of a single particle are replaced with new values. The most challenging part of the MCMC step is ensuring that the samples obtained realistically represent the desired distribution. It is known that convergence of the Markov chain fails for posteriors that are not proper, which can happen whenever the prior is improper (e.g., uniform density with infinite bounds) or non-identifiable parameters exist in the model [68]. In these situations, neither the prior assumptions nor the likelihood that represents the experimental data sufficiently constrain the posterior distribution. As such, the convergence properties of SMC and MCMC methods may improve considerably by resolving parameter identifiability issues before running the algorithm [69].

### Extensions to optimal experiment design

The selection of optimal conditions for conducting experiments (e.g., measurement times, initial conditions, and time-varying input profiles) is important for ensuring maximum information is extracted from the observations, especially when the experiments are expensive and time-consuming to perform. For example, it may be useful to change the feed rate or the measurement times in the considered case study so that the data ensures tight parameter estimates are obtained. Optimal experiment design (OED) has been extensively studied in the classical framework wherein the design criteria are defined as some scalar function of the Fisher information matrix (FIM) [70, 71]. More recently, OED has been tackled from a fully Bayesian perspective that replaces the approximated classical design criteria with an *expected utility* function that is rigorously chosen from a decision-theoretic point of view [72–74].

The nsPCE surrogate models could be used to efficiently evaluate classical or Bayesian design criteria at any fixed experimental condition. However, the parameter space decomposition depends strongly on the experiment, such that separate surrogates need to be constructed for all experiments of interest. This is not a major challenge when only a small number of experiments are considered, but may become intractable for continuous design spaces. Developing efficient procedures for both classical and Bayesian OED in genome-scale DFBA models is an important area for future research. One possible direction is to treat the experiment design variables as parameters when constructing the surrogate model, as suggested in [75] for global PCE. It would be interesting to see how well nsPCE can handle these additional dimensions, since the model responses would likely be highly sensitive to the design variables.

## Supporting information

S1 Fig Comparison of model predictions and synthetic data for the *E. coli* case study. The model predictions, shown with solid lines, were obtained by integrating the DFBA model with the maximum a posteriori (MAP) estimates of the parameters, which correspond to the mode of the posterior density. The ‘x’ marks represent synthetic data generated by corrupting model predictions for the true (unknown) parameters with randomly generated noise.
(PDF)

S2 Fig Parity plots for nsPCE surrogate models for synthetic metabolic network. The rows correspond to the extracellular substrate and product concentrations while the columns correspond to the various time points of interest. The *x*-axis represents the exact value of the model while the *y*-axis represents the surrogate model predictions. The blank plots represent quantities of interest with variance significantly lower than the tolerance.
(PDF)

S1 Table Nominal substrate uptake parameters for *E. coli* DFBA model. Parameter values taken from [8]. Uncertainty in the parameter estimates was not quantified. We assume the uncertainty in these estimates is uniformly distributed around ±10% of the nominal parameter values, which leads to fairly large variability in the predicted extracellular behavior.
(PDF)

S2 Table Relative mean square error estimates for nsPCE surrogate models for *E. coli* case study. A total of 1200 DFBA simulations were sequentially generated to train all nsPCE surrogate models to meet the specification *ε*_*target*_ = 10^−3^. The RMSE values were computed using a validation set of 10,000 DFBA simulations. An entry of 0.0 corresponds to quantities of interest with variance below *ε*_*target*_.
(PDF)

S3 Table Hierarchy of objectives for synthetic metabolic network. The FBA problem was formulated as a linear program with multiple objectives that are optimized based on the priority list specified in this table. This approach is able to ensure that the FBA problem is feasible for all simulation times and that the exchange fluxes are unique. More information on this strategy can be found in [10].
(PDF)

S4 Table Uncertain parameter bounds for synthetic metabolic network. The uncertainty ranges are based on the nominal values presented in [31, Chapter 8]. The parameters are either related to the substrate uptake kinetics, the initial conditions, or the stoichiometric coefficients of the reactions. For the latter, we selected coefficients that are likely to be inferred from experimental data in real applications.
(PDF)

S5 Table Simulated experimental data for synthetic metabolic network. This data was used to estimate the parameters in the synthetic case study, which was obtained by simulating the DFBA model with true (unknown) parameters and then adding randomly generated noise. The noise was assumed to be Gaussian with standard deviation shown in row labeled ‘STDEV’.
(PDF)

S1 Text Summary of methods for simulating DFBA models. (PDF)
