# Supplementary material for: Fast uncertainty quantification for dynamic flux balance analysis using non-smooth polynomial chaos expansions
Source: PLoS Comput Biol. 2019 Aug 30;15(8):e1007308. doi: 10.1371/journal.pcbi.1007308 (PMC6742419; doi:10.1371/journal.pcbi.1007308)
Supplement: S1 Fig — The model predictions, shown with solid lines, were obtained by integrating the DFBA model with the maximum a posteriori (MAP) estimates of the parameters, which correspond to the mode of the posterior density. The ‘x’ marks represent synthetic data generated by corrupting model predictions for the true (unknown) parameters with randomly generated noise. (PDF) [file pcbi.1007308.s001.pdf]

Supporting information: S1 Fig.

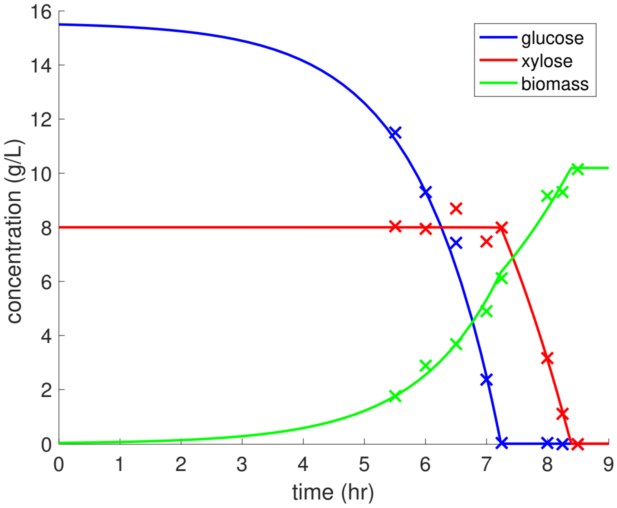

S1 Fig. Comparison of model predictions and synthetic data. The model predictions, shown with solid lines, were obtained by integrating the DFBA model with the maximum a posteriori (MAP) estimates of the parameters, which correspond to the mode of the posterior density. The 'x' marks represent synthetic data generated by corrupting model predictions for the true (unknown) parameters with randomly generated noise.
