# Supplementary material for: Fast uncertainty quantification for dynamic flux balance analysis using non-smooth polynomial chaos expansions
Source: PLoS Comput Biol. 2019 Aug 30;15(8):e1007308. doi: 10.1371/journal.pcbi.1007308 (PMC6742419; doi:10.1371/journal.pcbi.1007308)
Supplement: S2 Fig — The rows correspond to the extracellular substrate and product concentrations while the columns correspond to the various time points of interest. The x-axis represents the exact value of the model while the y-axis represents the surrogate model predictions. The blank plots represent quantities of interest with variance significantly lower than the tolerance. (PDF) [file pcbi.1007308.s002.pdf]

Supporting information: S2 Fig.

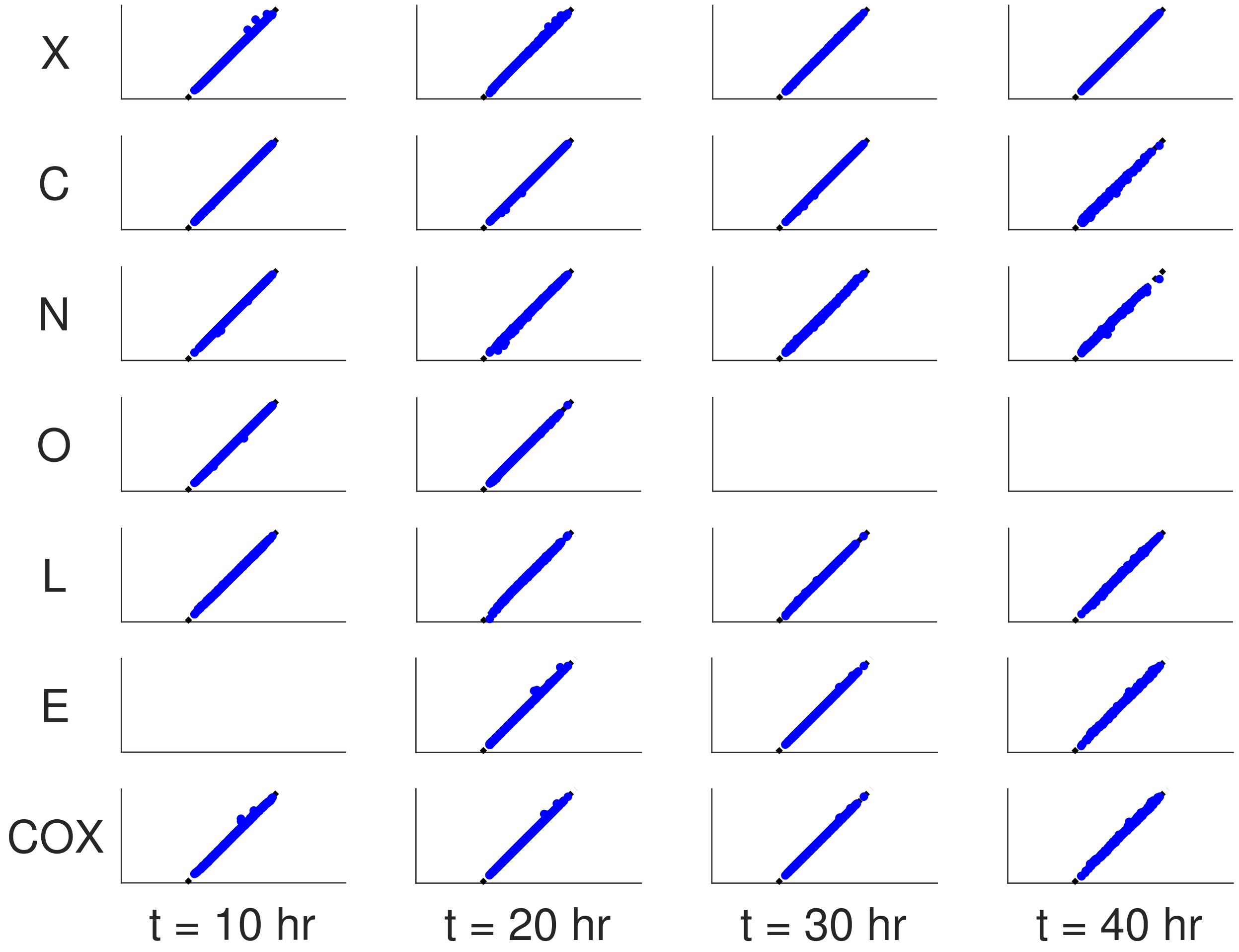

S2 Fig. Parity plots for nsPCE surrogate models for synthetic metabolic network. The rows correspond to the extracellular substrate and product concentrations while the columns correspond to the various time points of interest. The x-axis represents the exact value of the model while the y-axis represents the surrogate model predictions. The blank plots represent quantities of interest with variance significantly lower than the tolerance.
