# Supplementary material for: Fast uncertainty quantification for dynamic flux balance analysis using non-smooth polynomial chaos expansions
Source: PLoS Comput Biol. 2019 Aug 30;15(8):e1007308. doi: 10.1371/journal.pcbi.1007308 (PMC6742419; doi:10.1371/journal.pcbi.1007308)
Supplement: S1 Table — Parameter values taken from [8]. Uncertainty in the parameter estimates was not quantified. We assume the uncertainty in these estimates is uniformly distributed around ±10% of the nominal parameter values, which leads to fairly large variability in the predicted extracellular behavior. (PDF) [file pcbi.1007308.s003.pdf]

Supporting information: S1 Table

Parameter	Value	Units
$u_{g,max}$	10.5	mmol/g/hr
K_g	0.0027	g/L
$u_{z,max}$	6	mmol/g/hr
K_z	0.0165	g/L
K_{ig}	0.005	g/L
$u_{o,max}$	15	mmol/g/hr
K_o	0.024	g/L

S1 Table. Nominal substrate uptake parameters for *E. coli* DFBA model. Parameter values taken from [1]. Uncertainty in the parameter estimates was not quantified. We assume the uncertainty in these estimates is uniformly distributed around $\pm 10\%$ of the nominal parameter values, which leads to fairly large variability in the predicted extracellular behavior.

References

[1] Hanly TJ, Henson MA. Dynamic flux balance modeling of microbial co-cultures for efficient batch fermentation of glucose and xylose mixtures. Biotechnology and Bioengineering. 2011;108:376–385.
