# Supplementary material for: Fast uncertainty quantification for dynamic flux balance analysis using non-smooth polynomial chaos expansions
Source: PLoS Comput Biol. 2019 Aug 30;15(8):e1007308. doi: 10.1371/journal.pcbi.1007308 (PMC6742419; doi:10.1371/journal.pcbi.1007308)
Supplement: S2 Table — A total of 1200 DFBA simulations were sequentially generated to train all nsPCE surrogate models to meet the specification εtarget = 10−3. The RMSE values were computed using a validation set of 10,000 DFBA simulations. An entry of 0.0 corresponds to quantities of interest with variance below εtarget. (PDF) [file pcbi.1007308.s004.pdf]

Supporting information: S2 Table

t (hr)	Glucose (g/L)	Xylose (g/L)	Biomass (g/L)
5.5	5.5×10^{-5}	0.0	8.2×10^{-5}
6.0	5.2×10^{-5}	0.0	7.1×10^{-5}
6.5	3.9×10^{-5}	3.8×10^{-4}	7.4×10^{-5}
7.0	3.0×10^{-4}	6.4×10^{-5}	8.0×10^{-5}
7.25	6.1×10^{-5}	5.8×10^{-5}	6.6×10^{-5}
8.0	0.0	2.9×10^{-4}	4.8×10^{-4}
8.25	0.0	2.3×10^{-4}	2.3×10^{-4}
8.5	0.0	5.8×10^{-4}	4.0×10^{-4}

S2 Table. Relative mean square error estimates for nsPCE surrogate models for *E. coli* case study. A total of 1200 DFBA simulations were sequentially generated to train all nsPCE surrogate models to meet the specification $\varepsilon_{target} = 10^{-3}$. The RMSE values were computed using a validation set of 10,000 DFBA simulations. An entry of 0.0 corresponds to quantities of interest with variance below ε_{target}.
