# Supplementary material for: Fast uncertainty quantification for dynamic flux balance analysis using non-smooth polynomial chaos expansions
Source: PLoS Comput Biol. 2019 Aug 30;15(8):e1007308. doi: 10.1371/journal.pcbi.1007308 (PMC6742419; doi:10.1371/journal.pcbi.1007308)
Supplement: S3 Table — The FBA problem was formulated as a linear program with multiple objectives that are optimized based on the priority list specified in this table. This approach is able to ensure that the FBA problem is feasible for all simulation times and that the exchange fluxes are unique. More information on this strategy can be found in [10]. (PDF) [file pcbi.1007308.s005.pdf]

Supporting information: S3 Table

1	Maximize v_X
2	Maximize v_L
3	Maximize v_{Ferm}
4	Minimize v_C
5	Minimize v_N
6	Minimize v_O
7	Minimize v_{OX}

S3 Table. Hierarchy of objectives for synthetic metabolic network. The FBA problem was formulated as a linear program with multiple objectives that are optimized based on the priority list specified in this table. This approach is able to ensure that the FBA problem is feasible for all simulation times and that the exchange fluxes are unique. More information on this strategy can be found in [1].

References

[1] Gomez JA, Höffner K, Barton PI. DFBAlab: A fast and reliable MATLAB code for dynamic flux balance analysis. BMC Bioinformatics. 2014;15:409.
