# Supplementary material for: Fast uncertainty quantification for dynamic flux balance analysis using non-smooth polynomial chaos expansions
Source: PLoS Comput Biol. 2019 Aug 30;15(8):e1007308. doi: 10.1371/journal.pcbi.1007308 (PMC6742419; doi:10.1371/journal.pcbi.1007308)
Supplement: S4 Table — The uncertainty ranges are based on the nominal values presented in [31, Chapter 8]. The parameters are either related to the substrate uptake kinetics, the initial conditions, or the stoichiometric coefficients of the reactions. For the latter, we selected coefficients that are likely to be inferred from experimental data in real applications. (PDF) [file pcbi.1007308.s006.pdf]

Supporting information: S4 Table

Name	Range	Units
$v_{max,C}$	$[1.39, 1.61] \times 10^{+0}$	mmol/g/hr
K_C	$[4.62, 5.38] \times 10^{-2}$	g/L
K_{iE}	$[1.39, 1.61] \times 10^{+1}$	g/L
$v_{max,N}$	$[2.31, 2.69] \times 10^{-1}$	mmol/g/hr
K_N	$[4.62, 5.38] \times 10^{-1}$	g/L
$v_{max,O}$	$[1.85, 2.15] \times 10^{+0}$	mmol/g/hr
K_O	$[1.11, 1.29] \times 10^{+0}$	g/L
v_{ATP}	$[1.67, 1.94] \times 10^{-1}$	mmol/gDW
X_0	$[0.93, 1.08] \times 10^{-2}$	gDW/L
C_0	$[1.39, 1.61] \times 10^{+1}$	g/L
N_0	$[2.78, 3.23] \times 10^{-1}$	g/L
O_0	$[0.93, 1.08] \times 10^{+0}$	g/L
$S_{C,X}$	$[3.70, 4.30] \times 10^{+0}$	–
$S_{N,X}$	$[4.62, 5.38] \times 10^{-1}$	–
$S_{ATP,X}$	$[1.39, 1.61] \times 10^{+0}$	–
$S_{ATP,OX}$	$[0.93, 1.08] \times 10^{+0}$	–
$S_{ATP,Ferm}$	$[0.93, 1.08] \times 10^{+0}$	–
$S_{ATP,L}$	$[1.85, 2.15] \times 10^{+0}$	–
$S_{OX,OX}$	$[0.93, 1.08] \times 10^{+0}$	–
$S_{OX,Ferm}$	$[1.85, 2.15] \times 10^{+0}$	–

S4 Table. Uncertain parameter bounds for synthetic metabolic network. The uncertainty ranges are based on the nominal values presented in [1, Chapter 8]. The parameters are either related to the substrate uptake kinetics, the initial conditions, or the stoichiometric coefficients of the reactions. For the latter, we selected coefficients that are likely to be inferred from experimental data in real applications.

References

[1] Gomez JA. Simulation, sensitivity analysis, and optimization of bioprocesses using dynamic flux balance analysis. Massachusetts Institute of Technology; 2018.
