# Supplementary material for: Fast uncertainty quantification for dynamic flux balance analysis using non-smooth polynomial chaos expansions
Source: PLoS Comput Biol. 2019 Aug 30;15(8):e1007308. doi: 10.1371/journal.pcbi.1007308 (PMC6742419; doi:10.1371/journal.pcbi.1007308)
Supplement: S5 Table — This data was used to estimate the parameters in the synthetic case study, which was obtained by simulating the DFBA model with true (unknown) parameters and then adding randomly generated noise. The noise was assumed to be Gaussian with standard deviation shown in row labeled ‘STDEV’. (PDF) [file pcbi.1007308.s007.pdf]

Supporting information: S5 Table

Time (hr)	X	C	N	O	L	E	COX
STDEV	0.01	0.20	0.01	0.025	0.01	0.05	0.05
10	0.0580	15.6867	0.2520	0.7901	0.0194	0.0065	0.1667
20	0.2642	13.4934	0.1418	0.1372	0.1138	0.1024	1.0144
30	0.4935	7.6274	0.0190	0.0010	0.2155	1.2284	3.4080
40	0.5270	0.5941	0.0010	0.0001	0.4550	2.7062	6.3618

S5 Table. Simulated experimental data for synthetic metabolic network. This data was used to estimate the parameters in the synthetic case study, which was obtained by simulating the DFBA model with true (unknown) parameters and then adding randomly generated noise. The noise was assumed to be Gaussian with standard deviation shown in row labeled 'STDEV.'
