# Supplementary material for: Fast uncertainty quantification for dynamic flux balance analysis using non-smooth polynomial chaos expansions
Source: PLoS Comput Biol. 2019 Aug 30;15(8):e1007308. doi: 10.1371/journal.pcbi.1007308 (PMC6742419; doi:10.1371/journal.pcbi.1007308)
Supplement: S1 Text — (PDF) [file pcbi.1007308.s008.pdf]

Supporting information: S1 Text. Summary of methods for simulating DFBA models.

DFBA models can be classified as ordinary differential equations with embedded optimization (ODEO) wherein the lower-level FBA optimization can either be a linear program (LP) or nonlinear program (NLP). The vast majority of DFBA simulation has focused on LP objectives, though NLP objectives were recently tackled in [1]. DFBA simulation strategies can be broadly categorized as: (i) static optimization approach (SOA) [2], (ii) dynamic optimization approach (DOA) [2], (iii) direct approach (DA) [3], and (iv) interior point reformulation (IPR) [4]. In SOA, a forward Euler scheme is used to integrate the system while the FBA is solved at each time step using a suitable solver. SOA is known to be inefficient, especially for stiff problems that require small time steps to ensure convergence, due to the fact that the FBA optimization must be repeatedly solved. DOA, on the other hand, discretizes the time horizon and then converts the DFBA model into a NLP problem; however, this approach cannot be easily applied to genome-scale metabolic networks due to the large number of variables and constraints that are introduced. DA directly includes the solver for the FBA in the right hand side evaulator $\mathbf{f}$. Thus, DA can take advantage of implicit ODE integrators that employ adaptive step sizes and error control, which reduces the number of integration steps when compared to SOA. The IPR method is based on the fact that the optimization problem in ODEO models can be replaced by their Karush-Kuhn-Tucker (KKT) optimality conditions, which can subsequently be relaxed using logarithmic barrier functions so that the DFBA model can be transformed into a set of implicit ODEs. However, IPR introduces a new source of error since the relaxed solution only converges to the true optimal solution as the barrier parameter approaches zero.

References

[1] Zhao X, Noack S, Wiechert W, von Lieres E. Dynamic flux balance analysis with nonlinear objective function. Journal of Mathematical Biology. 2017;75:1487–1515.

[2] Mahadevan R, Edwards JS, Doyle III FJ. Dynamic flux balance analysis of diauxic growth in Escherichia coli. Biophysical Journal. 2002;83:1331–1340.

[3] Höffner K, Harwood SM, Barton PI. A reliable simulator for dynamic flux balance analysis. Biotechnology and Bioengineering. 2013;110:792–802.

[4] Scott F, Wilson P, Conejeros R, Vassiliadis VS. Simulation and optimization of dynamic flux balance analysis models using an interior point method reformulation. Computers & Chemical Engineering. 2018;119:152–170.
